# Site-specific N^6^-methyladenosine fine tunes CTCF/cohesin-defined enhancer-promoter interactions and oncogenic transcription program

**DOI:** 10.21203/rs.3.rs-10010180/v1

**Published:** 2026-08-14

**Authors:** Huacheng Luo, Karina Hamamoto, Jingwen Song, Tsz Kan Fung, Songtao He, Julia Lesperance, Ying Li, MingJiang Xu, Honglin Chen, Yi Qiu, Chi Wai Eric So, Suming Huang

**Affiliations:** 1 Division of Pediatric Hematology & Oncology, Department of Pediatrics, Pennsylvania State University College of Medicine, Hershey, PA 17033, USA; 2 Leukaemia and Stem Cell Biology Group, School of Cancer and Pharmaceutical Science, King’s College London, London SE5 9NU, UK; 3 Department of Molecular Medicine, The University of Texas Health Science Center at St Antonio, St Antonio, TX78229, USA; 4 Department of Microbiology, Laboratory Block, Li Ka Shing Faculty of Medicine, The University of Hong Kong, Hong Kong SAR, China; 5 Department of Cellular and Molecular Physiology, Pennsylvania State University College of Medicine, Hershey, PA 17033, USA; 6 Penn State Cancer Institute, Pennsylvania State University College of Medicine, Hershey, PA 17033, USA; 7 Current address: Zhejiang Cancer Hospital, Hangzhou Institute of Medicine, The Chinese Academy of Sciences, Hangzhou, Zhejiang 310022, China; 8 These authors contributed equally; 9 Lead contact

**Keywords:** m^6^A modification, *HOTTIP* lncRNA, CTCF/cohesin, TADs and enhancer/promoter loops, transcription regulation, MLL rearrangement, acute myeloid leukemia

## Abstract

N^6^-methyladenosine (m^6^A) of RNAs plays an important role in RNA stability and gene expression. However, it remains unknown how site-specific m^6^A on individual RNA is decoded to regulate chromatin architecture and transcriptional reprogramming. Here, we show that m^6^A modification of *HOTTIP* lncRNA is highly enriched in acute myeloid leukemia (AML) patients carrying *MLL*-rearrangement (*MLLr*^+^) or *NPM1*^*C*+^-mutation and regulates RNA-dependent CTCF/cohesin function. Site-specific removal/blockage of m^6^A on *HOTTIP* by CRISPR-dCas13-ALKBH5 or steric ASO blockage disrupts *HOTTIP*-associated R-loop formation and CTCF occupancies at *HOTTIP*/CTCF co-occupied TAD boundaries, leading to impaired CTCF-defined enhancer/promoter loops and *HOTTIP*-driven stem-cell-like transcription programs. Functionally, site-specific removal of m^6^A on *HOTTIP* impairs leukemic-stem-cell initiation/proliferation that mitigates *MLLr*^+^-driven AML severity. Targeted recruitment of RAD21 to the erased *HOTTIP* m^6^A site partially rescues CTCF-mediated enhancer/promoter interactions and *HOTTIP*-driven transcription, reversing the m^6^A depletion-associated antileukemic effects. Our data reveals a direct mechanistic interplay between site-specific RNA m^6^A modification and CTCF/cohesin-driven chromatin architecture to drive oncogenic transcription networks and leukemogenesis.

## INTRODUCTION:

N^6^-methyladenosine (m^6^A) is one of the most abundant RNA modifications in eukaryotic cells and is dynamically regulated by a methyltransferase complex comprising METTL3, METTL14, WTAP and RBM15 and demethylases such as ALKBH5 and FTO ^1-4^. Functionally, m^6^A-modified transcripts are recognized by reader proteins, including members of the YTH domain family (YTHDC and YTHDF proteins) and the IGF2BP family ^5,6^. Dysregulation of m^6^A regulatory proteins, leading to globally altered m^6^A deposition and recognition, has been implicated in normal hematopoiesis and acute myeloid leukemia (AML) ^7-11^.

Dynamic m^6^A within eukaryotic transcriptomes has been widely implicated in RNA metabolism, including RNA nuclear export, translation, degradation & stability, and splicing ^1,2^. In addition to the canonical roles in RNA metabolism, Recent studies have shown that RNA m^6^A directly regulates transcription elongation and termination via the function of METTL3 ^12,13^. Despite the RNA m^6^A roles in transcription, emerging studies have explored links between these epitranscriptomic marks and epigenetic gene regulation, including DNA and histone modifications mediated by m^6^A reader proteins ^14-16^. Furthermore, RNA m^6^A regulates DNA damage response, thereby playing a potential role in maintaining genome stability^17,18^. Notably, m^6^A of mRNA and enhancer RNA (eRNA) has been shown to facilitate YTH *N*^*6*^-methyladenosine RNA binding protein C1 (YTHDC1) and YTHDF2 mediated-nuclear condensation, suggesting the potential role of m^6^A in nuclear organization ^19-21^. Although it was shown that co-transcriptional deposition and blockage of m^6^A by deletion of METTL3 or YTHDC1 on chromosome-associated regulatory RNAs modulate chromatin accessibility and transcription ^22^, the direct causative role of m^6^A modification of RNA in modulating chromatin architecture and gene regulatory networks remains unknown as globally depletion of m^6^A writer or reader may result in indirect secondary effects on chromatin regulator gene expression. Thus, whether and how m^6^A modification at specific sites on individual RNAs contributes to chromatin architecture and gene regulation remains largely unknown.

CCCTC-binding factor (CTCF) has emerged as a master regulator of mammalian genome organization ^23-25^. CTCF/cohesin plays a central role in defining topologically associated domain (TAD) boundaries and constraining long-range interactions of DNA regulatory elements within the defined TADs ^23,26^. In addition, CTCF-binding sites (*CBSs*) located within TADs may facilitate and stabilize enhancer-promoter interactions to control cell type-specific transcription and cell identity^27^. Both CTCF and cohesin bind RNAs to facilitate their function in defining chromatin architecture ^28,29^, which may contribute to their functional versatility. However, it still remains unclear whether and how ubiquitously expressed CTCF achieves highly context-specific regulatory functions in gene expression by regulating TAD organization or enhancer-promoter communications.

Recently, we demonstrated that *HOTTIP*, a 5’ HOXA locus associated long non-coding RNA (lncRNA), is specifically up-regulated in MLL rearranged (*MLLr*^+^) AML ^30^, which is characterized by stem-cell-like transcriptional signature and correlates with high relapse rates and poor long-term survival in AML patients ^31,32^. Aberration of *Hottip* facilitates hematopoietic stem cell (HSC) self-renewal leading to AML-like disease in mice by regulating oncogenic TAD organization and leukemogenesis^30^. *HOTTIP* lncRNA rewires CTCF-driven TADs by co-occupied a subset of *CBSs* through formation of R-loop structure that regulate leukemic transcription program, including HOX-associated oncogenes and Wnt target genes, to promote and maintain the leukemic phenotype of *MLLr*^+^ and *NPM1*^*C+*^-driven AML ^30,33^. However, the molecular determiner(s) that regulates *HOTTIP* lncRNA oncogenic activity in *MLLr*^+^ AML remains unexplored.

Here, we demonstrate that two m^6^A modification sites of *HOTTIP* lncRNA are highly enriched in AML patients carrying *MLL*-rearrangement (*MLLr*^+^) or *NPM1*^*C+*^ mutation that not only positively correlates with *METTL3/METTL14* expression levels, but also physically interact with the m^6^A writers and readers. Site-specific removal of specific m^6^A from *HOTTIP* by guide RNA-mediated CRISPR-dCas13 targeted ALKBH5 or steric blockage of specific m^6^A site by anti-sense oligonucleotide (ASO) impairs its interactions with CTCF/cohesin complex and R-loop regulators that reduced *HOTTIP*-associated R-loop formation and co-occupancies at CTCF/cohesin boundaries, leading to disruption of CTCF-defined TAD and enhancer/promoter loops, inhibition of *HOTTIP*-driven leukemic transcription programs, and mitigation of AML disease progression in xenotransplanted/PDX mouse models. Precise restoration of RAD21 to the defective m^6^A site partially rescues leukemic transcription programs and AML disease progression. Thus, our study reveals a causative role of site-specific epitranscriptomic m^6^A marks in driving CTCF/cohesin-mediated genome organization and enhancer-promoter communications that determine *HOTTIP*-mediated oncogenic transcription circuits.

## RESULTS

### HOTTIP associates with m^6^A modifiers/readers and is highly enriched in m^6^A modification in AML patients.

We reported that *HOTTIP* lncRNA associates with CTCF/cohesin complex by unbiased *HOTTIP* chromatin isolation by RNA purification in conjunction with liquid chromatography tandem mass spectrometry (ChIRP-LC-MS/MS) identification ^33^. In addition, *HOTTIP*-ChIRP-MS specifically identified m^6^A methyltransferases, METTL3/METTL14 and m^6^A modification readers, YTHDF2, as strong *HOTTIP* partners (Figure S1A), suggesting that *HOTTIP* RNA may be m^6^A modified in *MLLr*^+^ AML cells. To confirm the specificity, we carried out ChIRP-WB and validated that *HOTTIP* lncRNA specifically associated with METTL3/METTL14, but not with histone deacetylase 1 (HDAC1) (Figure 1A) in *HOTTIP*-expressed MOLM13 AML cells. Furthermore, we *in vitro* transcribed human *HOTTIP* RNA, methylated with or without purified METTL3/METTL14 complex, and then incubated with MOLM13 nuclear extract to pull down *HOTTIP*-associated protein complexes (Figure 1B, Left). Compared to negative control GAPDH, YTHDF2, CTCF, RAD21, and DHX9 are specifically pulled down by m^6^A methylated *HOTTIP*, while EEF1A1 interacts with both methylated and unmethylated *HOTTIP* (Figure 1B), suggesting that m^6^A methylation is essential to mediate *HOTTIP* interactions with CTCF/cohesin complex and R-loop regulator, DHX9.

Next, we analyzed the RNA-seq data in the TCGA-LAML and TARGET-AML datasets for *METTL3* levels and its correlation with *HOTTIP* expression in patients carrying *MLLr*^+^/*NPM1*^*C+*^. Compared to healthy individuals (n=18) and *NPM1*/*MLL1* wild-type (WT) AML patients (n=26), AML patients carrying *NPM1*^*C*+^ or *MLLr*^+^ (n=30) exhibited significantly elevated levels of both *METTL3* and *HOTTIP* RNA (Figure S1B). Expression of *HOTTIP* lncRNA is positively correlated to *METTL3* expression with coefficient score of 0.2045 in patients carrying *MLLr*^+^/*NPM1*^*C+*^, but not in patients with WT *MLL1* and *NPM1* that show very weak correlation coefficient (0.076) (Figure S1C). Interestingly, patients with high expression of *METTL3*/*METTL14* have a significantly shortened survival as compared to patients with lower expression, consistent with what we saw in AML patients with elevated *HOTTIP* expression ^30,34^. Thus, METTL3/METTL14-mediated m^6^A of *HOTTIP* may be involved in AML patient prognosis.

To further examine whether *HOTTIP* RNA is m^6^A methylated and precisely map the sites of modification, we carried out the m^6^A enhanced crosslinking immunoprecipitation in combination with deep sequencing (m^6^A eCLIP-seq) in *MLLr*^+^ MOLM13 cells. We identified five enriched m^6^A sites (G1, G2, G3, G4, and G5) within the exon 3 of *HOTTIP* lncRNA in MOLM13 cells (Figure 1C). To unbiasedly validate that m^6^A modifications are specifically enriched in *MLLr*^+^/*NPM1*^*C+*^ AML patients, we performed m^6^A meRIP-seq comparing one MLL/NPM1 WT AML patient, one AML patient carrying *MLLr*^+^, and one AML patient carrying *NPM1*^*C+*^ mutation. m^6^A is indeed only enriched at G2 and G3 sites of *HOTTIP* lncRNA containing canonical m^6^A modification motif, AAC/AGGACU, in patients carrying *MLLr*^+^ or *NPM1*^*C+*^ mutation (Figure 1D). Furthermore, we validated that m^6^A modifications at G2 and G3 sites were significantly elevated in *MLLr*^+^/*NPM1*^*C+*^ AML patients (n=11) as compared to that of MLL/NPM1 WT AML patients (n=7) by carrying out m^6^A CLIP-qPCR (Figure 1E). In contrast, there is no difference in m^6^A modification at G0 site of exon 2 that shows low level m^6^A modification (Figures 1C, 1E). Indeed, full-length *HOTTIP* secondary structure analysis revealed that G2 and G3 m^6^A sites are located in a close proximity and away from other three identified m^6^A sites (Figure S1D). Thus, our data suggest that two m^6^A modification sites, G2 and G3, of *HOTTIP* may play an important role in *MLLr*^+^/*NPM1*^*C+*^-driven pathogenesis.

### The m^6^A modification of HOTTIP affects neither HOTTIP RNA expression nor stability.

m^6^A modifications of eukaryotic transcriptomes have largely been thought to be implicated in RNA metabolism^1,2^. We therefore examined the role of two patient-enriched m^6^A sites, G2 and G3 (as indicated in Figures 1D/E) in *HOTTIP* lncRNA expression and stability. To elucidate site-specific function of *HOTTIP* m^6^A modification, we employed CRISPR-dCas13-ALKBH5 fusion targeted by guide RNAs specifically to G2, or G3 m^6^A modified site of *HOTTIP* as comparing to no sgRNA (single guide RNA) control (dALK-Ctrl) in MOLM13 cells (Figure 2A). CRISPR-Cas13 is a flexible platform to target RNA for functional study in mammalian cells ^35^ and ALKBH5 is RNA demethylase that erases m^6^A from RNA ^36^. m^6^A eCLIP-seq assays of dCas13-ALKBH5 targeted MOLM13 cells revealed that targeting dCas13-ALKBH5 to G2 or G3 (dALK-G2 or dALKG3) site specifically erased m^6^A modification at specific m^6^A modified site of *HOTTIP* lncRNA without affecting neighboring m^6^A modification as compared to dALK-Ctrl (Figure 2B). In contrast, targeting dCas13-ALKBH5 to G2 or G3 site did not significantly alter m^6^A modifications in *HOXA* locus, nor genome-wide m^6^A modification patterns (Figure 2C, S2A). Thus, dCas13-ALKBH5 specifically erases m^6^A modification on targeted site. Given that m^6^A modification plays a critical role in RNA metabolism ^1,37,38^, we examined whether erase of m^6^A modification affects *HOTTIP* transcripts or stability. Indeed, removal of m^6^A modification from G2, or G3 site affects neither mature and nascent *HOTTIP* RNA transcripts (Figure 2D), nor *HOTTIP* RNA stability (Figure S2B), suggesting that m^6^A modification in G2 or G3 site of *HOTTIP* does not affect its RNA metabolism.

### Erasing m^6^A modification at specific sites of HOTTIP Impairs MLLr^+^ AML LSC proliferation and prolongs survival of transplanted AML mouse models.

Given that *HOTTIP* acts as an oncogene to promote leukemia stem cell (LSC) growth ^30^, we then assessed the effects of *HOTTIP* m^6^A erase on leukemic cell growth and viability. Removal of m^6^A at G2 or G3 site consistently and significantly reduced leukemic cell proliferation as compared to dALK-Ctrl (Figures 2E, S2C). Colony forming assay further validated that removal of m^6^A modification at G2 or G3 site of *HOTTIP* significantly reduced colony size and number due to inhibiting leukemic cell proliferation and initiation (Figures 2F, S2D). Furthermore, cell cycle analysis revealed that removal of m^6^A from either G2 or G3 site of *HOTTIP* blocked cells in G1 phase and reduced G2/M phases (Figure S2E), indicating that site-specific m^6^A of *HOTTIP* is involved in AML cell proliferation by regulating cell cycle progression.

Next, we examined whether removal of m^6^A modification at G2 or G3 site of *HOTTIP* affects AML cell leukemogenesis *in vivo*. To test this possibility, we transplanted 5 × 10^5^ Ctrl vs. dALK-Ctrl, dALK-G2, or dALK-G3 MOLM13 cells into irradiated NSG mice (Figure S2F). All mice transplanted with Ctrl or dALK-Ctrl MOLM13 AML cells carrying *MLLr*^+^ died around 46 days post-transplantation, while mice receiving cells erased m^6^A modifications at either G2 or G3 site of *HOTTIP* survived more than 56 days by reducing donor leukemia cell penetration and splenomegaly (Figures 2G, S2G, S2H). In addition, fluorescence-activated cell sorting (FACS) assay of recipients at 40 days post-transplantation revealed that the human CD45^+^ (hCD45^+^) cell chimerism was significantly reduced in mice receiving MOLM13 cells erased m^6^A modifications at either G2 or G3 site of *HOTTIP* (Figures 2H, S2H). Thus, site-specific removal of m^6^A modifications from *HOTTIP* lncRNA mitigated human leukemic blast in recipient mice.

Furthermore, m^6^A modifications of *HOTTIP* were also erased in two primary AML cells (DJ and UPN002) carrying *MLLr*^+^ obtained from AML patients by CRISPR-dCas13-ALKBH5 fusion targeting. We then test leukemia stem cell (LSC) initiating frequencies by long-term culture initiating cell assay (LTC-IC) in primary patient AML cells, DJ and UPN002, comparing Ctrl, dALK-Ctrl, dALK-G2, or dALK-G3. LT culture leukemic initiating cell frequencies of *HOTTIP* G2 or G3 m^6^A removed primary AML samples were significantly inhibited (Figures 2I, S2I). Furthermore, 2 × 10^5^ dALK-Ctrl, dALK-G2, dALK-G3, or in combination of both dALK-G2/G3 primary AML patient cells (DJ) were transplanted into irradiated NSG mice. Mice received dALK-Ctrl began to get sick and die around 75 days post-transplantation, while mice received either erasing m^6^A modifications at G2 and/or G3 site of *HOTTIP* significantly prolonged survival (Figures 2J, S2J). Consistently, loss of m^6^A modifications at G2 or G3 site of *HOTTIP* significantly increased survival of both *MLLr*^+^ AML cell line xenograft and PDX mouse models. Taken together, site-specific removal of m^6^A modification(s) from *HOTTIP* lncRNA reduced the AML penetration and leukemic burden *in vivo*.

### Erase of m^6^A modification at specific sites of HOTTIP impairs MLLr target gene transcription.

To investigate mechanism by which m^6^A of *HOTTIP* regulates *MLLr*^+^-driven leukemogenesis, we carried out RNA-seq compared among Ctrl, dALK-Ctrl, dALK-G2, and dALK-G3 MOLM13 AML cells. Transcriptomic profile revealed that dALK-G2 or dALK-G3 downregulated 81 or 258 genes, respectively, while dALK-Ctrl behaved like Ctrl MOLM13 cells (Figure 3A). Removal of m^6^A modifications of *HOTTIP* impaired the transcription not only HOXA, Wnt/β-catenin target genes, and homeotic oncogenes driven by *MLLr*^+^, but also many genes important for HSC function and leukemogenesis (Figures 3A, 3B, S3A). Gene ontology (GO) analysis revealed that genes involved in RNA Pol II transcription regulation, chromatin, homeobox, and Wnt signaling pathway were coordinately downregulated in dALK-G2 and dALK-G3 MOLM13 cells (Figure 3C). To validate the specificity of CRISPR-dCas13-mediated site-specific m^6^A erasing and to rule out the potential direct competition with m6A reader and CTCF binding due to the size of dCas13-ALKBH5 protein, we designed the steric blocking ASOs against G3 m^6^A modification motif, which were fully modified with 2’-O-Methoxyethyl (2'MOE) bases and phosphorothioate (PS) bonds to enhance the stability and RNA hybridization affinity of ASOs, as well as incorporated with a fluorescent dye Cy5 at 3’ end to monitor transfection efficiency. ASOs can bind to an RNA functional domain to sterically block proteins or other nucleic acids from binding at that site. We then transfected negative control (NC) ASO and steric blocking ASO targeting G3 m^6^A site into MOLM13 AML cells. Comparing to negative control (NC) ASO, m^6^A eCLIP-seq demonstrated that the G3 ASO specifically blocked m^6^A modification at G3 site of *HOTTIP* (Figure S3B). RNA-seq analysis revealed that steric blocking ASO targeting G3 m^6^A site of *HOTTIP* significantly reduces transcription of *HOTTIP* targets, including posterior HOXA genes, *MEIS1*, *PBX3*, *IRF8*, *CTNNB1*, and its target, *MYC*, without affecting *HOTTIP* transcript level (Figures 3D, 3E, S3C-S3D) in *MLLr*^+^ MOLM13 AML cells. Gene set enrichment analysis (GSEA) revealed that gene expression hallmarks involved in MYC targets, Wnt/β-catenin signaling, apoptosis, and G2M checkpoint were perturbed by steric blocking ASO targeting G3 m^6^A site of *HOTTIP* (Figure S3E) that is consistent with the signature transcriptome profiles of enzymatic site-specific removal of *HOTTIP* m^6^A modification (Figures 3A-C) and *HOTTIP* KO^30^.

Furthermore, steric blocking ASO targeting G3 m^6^A site significantly reduced proliferation of MOLM13 cells as compared to NC ASO (Figure 3F). Comparing to dALK-G3+ASO-NC positive control, steric blocking ASOs targeting G3 m^6^A exhibited compatible inhibition of *HOTTIP* target expression and cell proliferation without affecting *HOTTIP* transcript level (Figures 3F, S3D). As a positive control, *HOTTIP* degradation-inducing (Gapmer) ASOs inhibited both *HOTTIP* and *HOTTIP* target expression (Figure S3F). Colony and cell cycle assays demonstrate that steric blocking ASO targeting G3 m^6^A site of *HOTTIP* significantly reduced colony size and number (Figure 3G) and blocked cell in G1 phase and reduced cell in G2/M phases (Figure 3H), consistent with GSEA analysis (Figure S3E) and enzymatically removal of *HOTTIP* m^6^A modification did (Figures 2E-F). Thus, site-specific m^6^A modifications of *HOTTIP* specifically control *HOTTIP*-driven oncogenic transcription programs and leukemia cell proliferation.

#### Erase of m^6^A modification at specific site disrupted HOTTIP interactions with CTCF/cohesin complex and R-loop sensors/regulators.

CTCF/cohesin plays an essential role in genome TAD integrity to define enhancer/promoter actions for cell type-specific transcription^27,39^. Given that *HOTTIP* interacts with CTCF/cohesin complex and R-loop regulators^33^, we further explored the regulatory actions of *HOTTIP* m^6^A modifications by carrying out ChIRP-MS to unbiasedly identify the *HOTTIP*-associated proteomic complexes that are affected by site-specific m^6^A modifications through comparing among Ctrl, dALK-Ctrl, dALK-G2, or dALKG3 MOLM13 AML cells. We confirmed that *HOTTIP* is associated with CTCF/cohesin, R-loop sensors/regulators, MLL/WDR5, m^6^A modifier/reader/eraser complexes (Figure 4A). Compared to Ctrl and dALK-Ctrl MOLM13 cells, *HOTTIP* ChIRP-LC-MS/MS revealed that elimination of m^6^A modification at either G2 or G3 site of *HOTTIP* specifically disrupted interactions of *HOTTIP*-associated proteomic complexes, such as CTCF/cohesin complex, m^6^A reader YTHDF2, R-loop sensors and regulators, DHX9, SRSF1, and SUPT16H etc., as well as MLL complex (Figure 4A), suggesting that m^6^A modifications of *HOTTIP* maybe involved in chromatin organization, and therefore, stem-cell-like homeotic transcription program. As a control, elimination of m^6^A of *HOTTIP* did not affect *HOTTIP* interactions with RPL28, C4A, SNRPB, and EEF1A1 (Figure 4A).

To confirm the specificity of *HOTTIP* m^6^A mediated effects on *HOTTIP* interacting partners, we carried out ChIRP-western blot and validated that removal of m^6^A modification at G2 or G3 site of *HOTTIP* specifically reduced *HOTTIP* interactions with CTCF, RAD21, DHX9, and YTHDF2 (Figure 4B). Furthermore, RNA immunoprecipitation also confirmed that m^6^A modifications of *HOTTIP* are critical for *HOTTIP* to interact with CTCF, RAD 21, DHX9, MCM3, and YTHDF2 (Figure S4A). Thus, m^6^A modifications of *HOTTIP* regulates *HOTTIP* interactions with CTCF/cohesin complex and R-loop sensors/regulators. Furthermore, we examined the effects of steric blocking ASO targeting G3 m^6^A site of *HOTTIP* on its interaction with CTCF, METTL3, or YTHDF2 by CLIP-qPCR. Comparing to negative control NC ASO, steric blocking ASO targeting G3 m^6^A site significantly disrupted interaction between *HOTTIP* lncRNA and CTCF/cohesin, METTL3 or YTHDF2 in MOLM13 cells (Figures 4C-4D). It appears that *HOTTIP* and CTCF interaction is in part mediated by YTHDF2, as antibody depletion of YTHDF2 reduced the interaction between *HOTTIP* and CTCF (Figures S4B-S4C). Thus, steric blocking ASOs validated that m^6^A modification of G3 site regulates *HOTTIP*’s interaction with m^6^A writer METTL3, reader YTHDF2, and genome organizer CTCF/cohesin.

#### Site-specific m^6^A modifications regulate HOTTIP RNA-dependent CTCF/cohesin boundary activities in AML genome.

Given that erase of m^*6*^A modifications of *HOTTIP* reduce its interactions with both CTCF/cohesin and R-loop regulators (Figure 4), we thought to further determine whether elimination of m^6^A modifications of *HOTTIP* could affect CTCF chromatin boundary activity and if it does so by regulating R-loop formation at *HOTTIP* co-occupied *CBSs*. To test this possibility, we performed CTCF HiChIP, sonicated DNA/RNA immunoprecipitation sequencing (sDRIP-seq), and ChIRP-seq comparing Ctrl and dALK-G3 MOLM13 AML cells. Consistent with loss of CTCF/cohesin and R-loop regulator interactions (Figure 4), integration analysis of CTCF binding from HiChIP and *HOTTIP* chromatin binding from *HOTTIP* ChIRP-seq revealed that although removal of m^6^A modification at G3 site of *HOTTIP* did not globally alter CTCF binding (Figure 5A, Top), loss of m^6^A modifications specifically reduced CTCF binding at intergenic regions or promoters of the *HOTTIP*/CTCF co-occupied sites (Intergenic and promoter regions) in AML genome (Figure 5A, Bottom). Furthermore, combined integrated analysis of these data with global R-loop patterns from sDRIP-seq data further indicated that R-loops were also reduced specifically in *HOTTIP* chromatin binding sites or *HOTTIP*/CTCF co-occupied sites in AML genome upon removal of m^6^A modification at G3 site of *HOTTIP* lncRNA (Figure 5B), consistent with the ChIRP-MS data that site-specific m^6^A modifications facilitate *HOTTIP* interactions with CTCF/cohesin and R-loop sensors/regulators (Figure 4).

CTCF/cohesin forms *HOTTIP*-dependent TAD boundaries in several loci critical for HSC/LSC self-renewal and AML leukemogenesis^33^. Consistent with altered stem-cell-like homeotic transcription program such as *CTNNB1*, *MYC*, *MEIS1*, *MECOM*, and posterior HOXA genes by erasing m^6^A modifications of *HOTTIP* (Figure 3A), Co-occupancies of CTCF, *HOTTIP* lncRNA, and R-loops at boundary *CBSs* of these hematopoietic/leukemogenic oncogene loci were impaired by erasing m^6^A modification at G3 site of *HOTTIP* lncRNA (Figures 5C-D, S5A-S5E). For example, removal of m^6^A modifications of *HOTTIP* reduced coordinated *HOTTIP* binding, R-loop formation, as well as CTCF binding in *CBS-u2* TAD boundary in the upstream of *CTNNB1* and in the promoter of *MYC* oncogene that are essential for forming AML specific TAD topology (Figures 5C-D, S5A-B). Furthermore, co-occupancies of *HOTTIP*, R-loop, and CTCF in *MEIS1*, *MECOM*, and *HOXA* TAD or sub-TAD boundaries were also impaired by the erase of m^6^A modifications of *HOTTIP* RNA (Figures S5C-E), again consistent with downregulation of these leukemic oncogenes and oncogenic pathways by removal of m^6^A modification at G3 site of *HOTTIP* (Figure 3A).

To validate that erase of m^6^A modification at G3 site of *HOTTIP* blocks interaction between *HOTTIP* and CTCF and R-loop formation at *HOTTIP*/CTCF co-occupied CTCF TAD boundary that resulted in reduced CTCF chromatin binding, we examined the effects of steric blocking ASO targeting G3 m^6^A site of *HOTTIP* on R-loop formation and CTCF/cohesin recruitment at known *CBS-u2* upstream TAD boundary of *CTNNB1* locus by sDRIP-qPCR and ChIP-qPCR. Comparing to negative control ASO-NC, steric blocking ASO targeting G3 m^6^A site significantly reduced R-loop formation at *CBS-u2* of *CTNNB1* locus (Figure S5F), but not in *RPL13* promoter, a *HOTTIP*-independent R-loop control (Figure S5G). Furthermore, both CTCF and RAD21 binding to the *CBS-u2* of *CTNNB1* and *MYC promoter* loci were significantly decreased, but not a negative control region in the exon of *MYC* (Figure S5H). Thus, site-specific m^6^A modifications of *HOTTIP* play critical role in regulating CTCF TAD boundary function, perhaps by modulating *HOTTIP* lncRNA associated R-loop formation.

#### Site-specific m^6^A modifications facilitate HOTTIP-dependent CTCF TAD integrity and CTCF-driven chromatin loops in AML genome.

Next, we test whether m^6^A modifications of *HOTTIP* controls CTCF-mediated TAD integrity and enhancer/promoter communications within CTCF defined TADs by carrying out HiC and CTCF HiChIP comparing Ctrl, dALK-G2, or dALK-G3 MOLM13 AML cells. Consistent with *HOTTIP* loss did not affect global CTCF binding in AML genome, erase of m^6^A modifications of *HOTTIP* reduces a small subset of CTCF binding in MOLM13 genome that consists of only 2.8% of total CTCF bindings (Figure S6A). Correlatively, removal of m^6^A modification at G3 site not only reduced CTCF loop intensity (Figure 6A), but critically disrupted 3773 CTCF-mediated loops (Figure S6B). a total of 17.0% of CTCF-mediated long-range chromatin loops were impaired (completely loss-of-loops) by removal of m^6^A modification at G3 site of *HOTTIP* lncRNA in the *MLLr*^+^ AML genome (Figure S6B). In particular, both CTCF bindings and CTCF-associated chromatin loops in the *MLLr*^+^ target gene loci, such as *CTNNB1*, *MYC*, *IRF8*, and *STAT3/5*, were markedly reduced (Figures 6B, S6C-D), consistent with transcriptome changes from RNA-seq analysis (Figure 3A). Although removal of m^6^A modification at G2 or G3 site does not affect global TAD structure (Figure S6E), Hi-C analysis comparing dALK-Ctrl, dALK-G2, or dALKG3 MOLM13 cells revealed that TAD and sub-TAD integrity at MLLr stem-cell-like target genes, *MYC* and *MECOM* loci, were markedly impaired upon removal of m^6^A modifications at G2 or G3 site of *HOTTIP* lncRNA (Figure 6C and S6F-S6G). Thus, site-specific m^6^A modification of *HOTTIP* regulates CTCF-driven TAD integrity and enhancer/promoter loops, perhaps through formation of R-loop at *HOTTIP*/CTCF co-occupied TAD boundaries.

To explore the mechanism underlying site-specific m^6^A removal mediated loss of CTCF-driven TADs and long-range loops, we performed ATAC-seq to examine changes in chromatin accessibilities comparing among Ctrl, dALK-G2, or dALK-G3 MOLM13 cells. Site-specific removal of m^6^A modifications of *HOTTIP* did not globally affect promoter chromatin accessibility in the AML genome (Figure 6D). However, consistent with reduction of CTCF-driven loops in *MLLr*^+^ target genes (Figures 6A-C, S6B-D), ATAC peaks were decreased in the promoters of *CTNNB1*, *MYC*, *IRF8*, and *STAT3/5* genes (Figure 6E, S6H), supporting that site-specific m^6^A modifications of *HOTTIP* regulate *HOTTIP*-dependent CTCF-driven long-range interactions and target gene regulatory circuits.

#### Targeted recruitment of RAD21 to the m^6^A erased G3 site of HOTTIP rescues CTCF-driven loops and leukemogenesis

Given that CTCF/cohesin are key genome regulators and associated with *HOTTIP* lncRNA in a m^6^A dependent manner (Figures 1B, 4), we next tested if specifically targeting RAD21 to m^6^A erased site of *HOTTIP* would rescue the effects of *HOTTIP* m^6^A loss by targeting CRISPR-dCas13-RAD21 to dALK-G3 cells that have expressed sgRNAs specifically targeting G3 m^6^A site of *HOTTIP*. RAD21 RIP confirmed that targeted expressing dCas13-RAD21 on G3 m^6^A-erased *HOTTIP* lncRNA partially restored *HOTTIP* and CTCF/cohesin complex interactions (Figure 7A) and expression of several key *HOTTIP* regulated homeobox and β-catenin target genes, such as *MYC* (Figure 7B, S7A). GO analysis revealed that RAD21 rescued upregulated genes are involved in hematopoiesis, cell death/migration, and cellular proliferation (Figure 7C). Furthermore, to explore the underlying mechanism, we tested whether restoration of cohesin at m^6^A erased G3 site of *HOTTIP* rescue CTCF mediated long-range chromatin loops required for homeotic oncogene activation as well, by carrying out CTCF HiChIP comparing Ctrl, dALK-G3, and dALK-G3+dALK-RAD21 *MLLr*^+^ MOLM13 cells. Consistent with the ability of dALK-G3+dALK-RAD21 to partially rescue CTCF interaction and target gene expression of G3 m^6^A modification-erased cells, HiChIP analysis revealed that restoration of RAD21 at m^6^A modification-erased G3 site of *HOTTIP* significantly rescued CTCF-driven long-range chromatin loops in AML genome, especially in the loci of *MYC* and *CTNNB1* (Figure 7D, S7B).

Functionally, restoration of RAD21 at m^6^A erased G3 site of *HOTTIP* partially rescued dALK-G3-VC transduced MOLM13 cell proliferation (Figure S7C). Colony forming assay revealed that removal of m^6^A modifications of *HOTTIP* consistently compromised colony forming ability including reducing both colony size and number due to inhibiting leukemic cell proliferation and initiation (Figures 7E, S7D comparing Ctrl and dALK-G3 VC), while restoration of RAD21 at m^6^A erased G3 site of *HOTTIP* largely rescued the colony forming ability of dALK-G3-VC transduced AML cells (Figures 7E, S7D). To test if restoration of RAD21 at m^6^A erased G3 site of *HOTTIP* rescues leukemogenesis *in vivo*, we transplanted 2 × 10^6^ Ctrl, dALK-G3-VC, and dALK-G3+dALK-RAD21 *MLLr*^+^ MOLM13 cells into irradiated NSG mice. All mice transplanted with Ctrl MOLM13 AML cells carrying *MLLr*^+^ died around 32 days post-transplantation, while most mice receiving cells erased m^6^A modifications of *HOTTIP* survived until end of xenograft experiment by reducing donor leukemia cell penetration and splenomegaly (Figures 7F, S7E-S7F). Fluorescence-activated cell sorting (FACS) assay of recipients at 24 days post-transplantation revealed that the human CD45^+^ (hCD45^+^) cell chimerism was significantly reduced in mice receiving dALK-G3 VC MOLM13 cells while the hCD45^+^ chimerism was significantly rescued in mice received dALK-G3+dALK-RAD21 *MLLr+* MOLM13 cells that restore cohesin at m^6^A erased G3 site of *HOTTIP* (Figures S7F). The effects became much profound when the RAD21 rescued animals die. Indeed, all mice received dALK-G3+dALK-RAD21 *MLLr+* MOLM13 cells with restored cohesin at m^6^A erased G3 site of *HOTTIP* largely rescued leukemia penetration and progression and died around day 43 evidenced by pale tibia bone, splenomegaly, and increased human CD45 leukemic blast in bone marrow and spleen at the time of the mice die (Figures 7F, S7G-S7H). Thus, *HOTTIP* site-specific m^6^A modification-dependent oncogenic gene regulatory networks and leukemogenesis are mediated by CTCF/cohesin-driven long-range chromatin loops.

## DISCUSSION:

RNAs including long-noncoding RNAs and RNA-binding proteins have been demonstrated to modulate CTCF-associated chromatin organization and transcriptional regulation during normal and disease development^33,40-42^. Furthermore, RNA-associated R-loop structure has been implicated in CTCF-directed genome topology^33,43-45^. Together, these studies highlight RNA as an increasingly important regulatory layer in nuclear organization. Despite these advances, a key conceptual gap remains in understanding how RNA m^6^A modification is decoded and integrated with architectural protein function like CTCF to shape higher-order genome organization in causative manner, as global manipulation of m^6^A reader, writer, or eraser often resulted in indirect secondary transcriptional effects. to interrogate direct causal regulatory action of specific m^6^A modification of RNA, we demonstrate that site-specific m^6^A on the oncogenic lncRNA *HOTTIP* is a key determinant of its oncogenic function through precisely editing these epitranscriptomic marks using dual strategies, dCas13-ALKBH5 -mediated demethylation and steric-blocking ASOs. Site-specific m^6^A modification regulates lncRNA driven R-loops and CTCF chromatin binding at key TAD boundaries, which in turn controls CTCF-driven enhancer-promoter interactions and MLLr^+^ associated stem-cell-like transcriptional programs. Notably, targeted recruitment of RAD21 to specific m^*6*^A erased site of *HOTTIP* partially rescued CTCF-driven enhancer-promoter loops and reversed antileukemic effects *in vivo*, supporting the new model that RNA-dependent CTCF genome organization and transcriptional regulation is regulated by site-specific m^6^A modification. Thus, we mechanistically demonstrated a new paradigm in which site-specific m^6^A modification provide another layer of regulation that fine-tunes CTCF/cohesin-driven TAD dynamics and long-range chromatin interactions required for stem-cell like homeotic/β-catenin oncogene expression.

Abundant coding and non-coding m^6^A RNA modifications have emerged a new layer of regulatory mechanism controlling the fates of the modified RNA molecules^1,46^, of which m^6^A modification canonically controls mRNA decay, stabilization and translation^38,46,47^. Therefore, dysregulation of m^6^A effectors including writers, METTL3/METTL14^7-10^, and erasers, ALKBH5 and FTO^48,49^, plays an essential role in oncogenesis including AML by globally perturbing oncogene or tumor suppressor expression patterns. However, no prior evidence has demonstrated that nucleotide-level targeting of a single m^6^A site can modulate leukemogenic transcriptional programs. Our study defines a site-specific causative epitranscriptomic regulation mechanism of an oncogenic lncRNA *HOTTIP*. Erase or blockage of hot-spot m^6^A modification sites of *HOTTIP* did not alter its expression, decay and stability (Figures 2D, 3E, and S2B), yet significantly impaired *HOTTIP*-directed stem-cell like gene chromatin and expression signatures. This strongly suggests that m^6^A directly regulates *HOTTIP*-driven gene regulatory network independent of its role in RNA metabolism.

It remains elusive whether m^6^A modification directly contributes to chromatin structure and epigenetic regulation of gene expression. Recent studies support a regulatory role for RNA m^6^A modification in transcription by modulating TET1 mediated DNA demethylation^15^ and pausing of RNA polymerase II (RNAPII)^12^. Furthermore, phase-separated transcriptional nuclear condensates are facilitated by RNA m^6^A modification and its reader YTHDC1^19,20^, underscoring its potential regulatory role in 3D genome organization and transcriptional activation. Co-transcriptional m^6^A modification on various chromosome-associated regulatory RNAs (carRNAs) has been shown to promote gene transcription by increasing chromatin accessibility^22^. Moreover, previous study has shown that m^6^A modification of the lncRNA Xist regulates transcriptional silencing through recruitment of the reader YTHDC1, supporting a local, RNA-specific m^6^A regulatory mechanism on gene expression ^50^. Here, we extend this paradigm by showing that specific targeting on the hotspot m^6^A sites on *HOTTIP* is sufficient to modulate CTCF-defined enhancer/promoter communications and associated stem-cell like homeotic/β-catenin oncogene expression, thereby contributing to leukemogenesis.

CTCF/cohesin complex is the fundamental building block of 3D genome structure^51-54^ and increasing evidence shows that their activity can be regulated by RNAs^28,29,55^. Direct interactions between CTCF and RNAs have been proposed^55-57^, supported by the presence of RNA-binding zinc finger domain of CTCF^28,29^ and identification of consensus RNA motif capable of interacting with CTCF in human cells^58^. Although evidence for direct RNA-CTCF interaction was challenged by *in vitro* purification studies of RNA-containing complex performed under denaturing condition^59^, our current data (Figures 1A-B and 4A-C) and recent reports ^33,40,60^ support that RNA-CTCF interactions are robustly identified under native or crosslinked RNA purification conditions, supporting an RNA-dependent CTCF/cohesin function. The discrepancy in different purification studies could be due to that RNA-CTCF interactions occur in large protein complexes or nuclear condensates where other RNA binding proteins (RBPs) may mediate their interaction. Indeed, we found that m^6^A modified *HOTTIP* specifically associates with YTHDF2 and CTCF/cohesin (Figures 1A-B and 4), reinforcing this RBP-dependent mechanism. Alternatively, it is also possible that RNA-CTCF interactions and function are context-dependent. For example, it was reported that RNA binding proteins-CTCF interactions are facilitated by the lncRNA Pantr1, which is progressively required during embryonic stem cells to neural stem cell differentiation^40^. Whether other RNA-binding proteins and effectors including other R-loop effectors or phase separation factors are also involved in facilitating this association warrants further investigations.

How specific m^6^A modification site of RNA modulates CTCF/cohesin-directed TAD formation and enhancer-promoter interactions for gene expression remains poorly understood. YTHDF2 is a canonical reader of m^6^A-modified RNA of which the YTH domain of YTHDF2 forms aromatic cage to recognize and bind m^6^A modified RNAs that facilitate RNA regulatory functions^61-63^. It is particularly interesting that YTHDF2 was shown to be the main reader of m^6^A modified R-loops, therefore m^6^A modification can potentially destabilize or promote R-loop formation via YTHDF2 recognition^17^. Consistently, our data suggests that YTHDF2 may be the key mediator of m^6^A-mediated *HOTTIP* function. Given that YTHDF2 is highly enriched with CTCF/cohesin complex in m^6^A modified *HOTTIP*-associated protein complex (Figures 1B and 4) and depletion of YTHDF2 destabilized the *HOTTIP*-CTCF interaction (figure S4C), it is conceivable that recruitment of CTCF/cohesin to m^6^A modified *HOTTIP* at the *HOTTIP* co-occupied TAD boundaries is mediated by the reader YTHDF2, which warrants further investigation. Another scenario is that YTHDF2 may facilitate additional R-loop effectors to modulate R-loop formation of m^6^A modified *HOTTIP* lncRNA, thereby enhancing *HOTTIP*-CTCF/cohesin co-occupancy at TAD boundaries and strengthening TAD topology and enhancer/promoter interactions in *HOTTIP* target genes. Nevertheless, our findings define a new mechanism in which site-specific m^6^A modification communicates with CTCF/cohesin complex to regulate RNA-dependent TAD dynamics and enhancer/promoter loops, likely through modulating YTHDF2-bound *HOTTIP* R-loops at key RNA-dependent CTCF TAD boundary sites.

MLLr^+^ AML is aggressive disease that is driven by leukemia stem cells and epigenetic dysregulation and associated with high relapse rates and poor long-term survival in patients^31,32^. Conventional treatment of this specific type of AML remains challenging due to inability of eliminating LSCs and frequent treatment related lineage skewing^64^. Although new target therapies, such as Menin inhibitor, show promising^65^, identifying m^6^A modification of *HOTTIP* as a molecular switch/determinant that modulates CTCF-mediated 3D leukemic genome organization and MLLr^+^-driven stem-cell-like transcription signature improves understanding of the molecular underpinning of MLLr^+^-driven leukemogenesis. Site-specific targeting critical m^6^A site of oncogenic lncRNA may provide new therapeutic strategies. Indeed, the ASO targeting m^6^A site showed specific blockage of site-specific m^6^A modification on *HOTTIP*, inhibiting MLL target gene transcription signature, and reducing leukemia cell proliferation (Figure 3 and S3), suggesting that targeting critical m^6^A site or its R-loop domain by ASO may be exploited to eliminate functional RNA-driven disease such as leukemia. However, efficiency and safety of ASOs used for leukemic treatment remains challenging and warrant further study.

## Materials and methods

### CONTACT FOR REAGENT AND RESOURCE SHARING

Further information and requests for reagents may be directed to and will be fulfilled by the lead contact, Dr. Suming Huang (shuang4@pennstatehealth.psu.edu)

### MATERIALS AVAILABILITY STATEMENTS

This study did not generate new unique reagents.

All unique/stable reagents generated in this study are available from the Lead Contact without restriction.

All unique/stable reagents generated in this study are available from the Lead Contact with a completed Materials Transfer Agreement.

### EXPERIMENTAL MODEL AND SUBJECT DETAILS

#### AML patient samples

The acquisition of primary MLLr^+^, NPM1c^+^, or MLLr^−^/NPM1^c−^ AML patient samples was approved by the Institutional Review Boards of the University of Florida and the Cancer Institute of the Pennsylvania State University College of Medicine in accordance with the Declaration of Helsinki.

#### Cell lines

HEK293T cells were cultured in DMEM supplemented with 10% bovine serum (FBS) and 1X penicillin/streptomycin, MOLM13 AML cells were cultured in RPMI 1640 supplemented with 10% FBS and 1X penicillin/streptomycin. All cells were maintained at 37°C with 5% CO_2_ in a humidified incubator.

### METHOD DETAILS

#### Generation of CRISPR-dCas13 mediated m6A depleted AML cells with catalytically dead dCas13-ALKBH5 system

To generate the depletion of specific HOTTIP m6A sites, dCas13-ALKBH5 vector was generated by subcloning pCMV-ALKBH5 fused DNA (Addgene # 134783) into the CRISPR-pCMV-dCas13 vector (Addgene #155367) with a XbaI-ClaI restriction fragment. dCas13 guide RNAs(gRNAs) targeting the different HOTTIP m6A sites (G2 and G3) were subcloned into pC0043-PspCas13b-crRNA (Addgene #103854) with BbsI (NEB) enzyme, which has constitutive gRNA expression under a U6 promoter, including dCas13-ALKBH5-gRNA-control (dALK-Ctrl), dCas13-ALKBH5-gRNA-2 (dALK-G2), dCas13-ALKBH5-gRNA-3 (dALK-G3) vectors. All designed gRNAs were subjected to MEGABLAST website (https://blast.ncbi.nlm.nih.gov/Blast.cgi) to avoid mismatch of unexpected RNAs in the human genome.

#### Rescue of *HOTTIP/RAD21* interaction in m6A depleted MOLM13 cells by dCas13-RAD21

To recover the interaction between HOTTIP and RAD21 in m6A depleted MOLM13 cells, dCas13-RAD21 was generated by subcloning full length of RAD21 from TFORF3187 (Addgene #144663) into dCas13d-BFP vector (Addgene#215720). dCas13 conjugating with RAD21 is transduced by lentiviral infection in dALK-G3. BFP positive dALK-G3 cells were sorted by BD FACS Aria SORP high-performance cell sorter (BD).

#### RNA isolation, quantitative RT-PCR, and RNA-sequencing

Total RNA was purified with the Quick-RNA Miniprep Kit (ZYMO Research) according to the manufacturer's instructions. For cDNA synthesis, 1 μg total RNA was used for reverse transcription using the High-Capacity cDNA Reverse Transcription Kit (Thermo Fisher Scientific #4387406) by following the manufacturer’s instructions. Then, cDNA was analyzed by quantitative PCR using the primers listed in table. GAPDH or ACTB was used as control and each reaction was run in triplicates.RNA sequencing libraries were prepared from 1 μg of total RNA using the NEBNext Ultra II Directional RNA Library Prep kit (E7765, New England Biolabs) following manufacturer instructions. Briefly, mRNA was purified using poly-T oligo beads and fragmented. Subsequently, first- and second- strand cDNA synthesis was performed, libraries were amplified, and samples were indexed. Library quality was assessed by Qubit and Agilent Bioanalyzer. Libraries were subjected to paired-end sequencing at a 150 bp length on an Illumina NovaSeq 6000. Sequence reads were trimmed and quality filtered using cutadapt (http://cutadapt.readthedocs.io, version 1.18) ^1^ and aligned to the human (hg19) genome using HISAT2 (version 2.2.1). FPKM (paired-end fragments per kilobase of exon model per million mapped reads) values were calculated using Cufflinks v2.2.1 and differential expression analysis was performed using Cuffdiff ^2^. The heatmap was generated using GraphPad Prism (version 10.3.1) based on z-score of the FPKM values ^3^. For differential analysis, we used an adjusted p-value < 0.01 or adjusted p-value<0.05 and ∣log2(fold change)∣ > 1. Gene set enrichment analysis (GSEA) was performed with gene sets obtained from the Molecular Signatures Database ^4^. GO analysis of differentially expressed (greater than 2 fold) genes was generated using Database for Annotation, Visualization and Integrated Discovery (DAVID) Bioinformatics Resources ^5^ and Gorilla ^6^. Sequence reads have been deposited in the NCBI GEO under accession number (GSE310379).

#### mRNA stability assay

The mRNA degradation experiments were performed as described previously with some modifications ^7^. Actinomycin D (Sigma-Aldrich) was added to AML cells at 2 μg/ml-5 μg/ml to assess RNA stability. In brief, 2 × 10^5^ cells were seeded in 6-well plates. After 48 h, cells were treated with a final concentration of 2 μg/ml-5 μg/ml actinomycin D for 0, 2, 4, 6, 8, or 10 h. After incubation for indicated time points, these treated and untreated cells were collected and RNA samples were extracted for reverse transcription and qPCR analysis. Half-life (t1/2) of mRNA was calculated using ln2/slope and including GAPDH for normalization.

#### shRNA-mediated METTL3 knockdown

MOLM13 AML cells were transduced with lentiviruses expressing shRNAs (shRNA KD1, 5′-GCTGCACTTCAGACGAATTAT-3′ [TRCN0000034718]; shRNA KD2, 5′-CGTCAGTATCTTGGGCAAGTT –3′ [TRCN0000034716]; and shRNA CTL, 5′-TTCTCCGAACGTGTCACGTT-3′; GE Healthcare). Selection of efficiently transduced cells was achieved by treatment with puromycin (2 μg/mL final concentration).

#### Cell Cycle Analysis

Ctrl and modified MOLM13 cells were harvested and washed with phosphate buffered saline (PBS). The washed cells were fixed by adding 70% ethanol drop wise to the pellet with vortexing and incubated overnight at 4°C. After fixation, cells were washed with PBS twice. The PBS washed cells were treated with the FxCycle^™^ PI/RNase Staining Solution (Thermo Fisher Scientific #F10797) and then incubated at 4°C for 30 min in the dark. Stained samples were proceeded on the NovoCyte Advanteon Flow Cytometer (Agilent), and cell cycle data analysis was performed using FlowJo program.

#### ASO and ASO transfection

The steric-blocking antisense oligonucleotides (ASOs) (Integrated DNA Technologies) targeted to the m6A sites were fully modified with 2′-O-methoxyethyl (2′MOE) bases and phosphorothioate bonds, which were also incorporated with a fluorescent dye Cy5 at the 3′ end to monitor transfection efficiency. The NC (non-targeting) ASO was used as a negative control that was not targeted to the human or mouse genome ^8^. Degradation ASOs (Gapmer ASOs) were fully modified with phosphorothioate bonds, and the first and last 5 bases in each ASO are 2′MOE modified. ASO transfections in MOLM13 cells were performed using the Lipofectamine 3000 Transfection Reagent (Thermo Fisher Scientific #L3000008) according to the manufacturer’s instructions.

For Steric-blocking ASOs:

NC 2′MOE: GCGACTATACGCGCAATATG

Sequence:

/52MOErG/*/i2MOErC/*/i2MOErG/*/i2MOErA/*/i2MOErC/*/i2MOErT/*/i2MOErA/*/i2MOErT/*/i2M OErA/*/i2MOErC/*/i2MOErG/*/i2MOErC/*/i2MOErG/*/i2MOErC/*/i2MOErA/*/i2MOErA/*/i2MOEr T/*/i2MOErA/* /i2MOErT/*/i2MOErG//3Cy5Sp/

ASO-G3 2’MOE: ACTGCCCAGTCCTGGTTTCC

Sequence:

/52MOErA/*/i2MOErC/*/i2MOErT/*/i2MOErG/*/i2MOErC/*/i2MOErC*/i2MOErC*/i2MOErA*/i2MO ErG*/i2MOErT*/i2MOErC*/i2MOErC*/i2MOErT*/i2MOErG/*/i2MOErG/*/i2MOErT/*/i2MOErT/*/i2MOErT/*/i2MOErC/*/i2MOErC//3Cy5Sp/

For Gapmer (degradation) ASOs (Bold indicates 2′MOE, all linkages are phosphorothioate bonds):

ASO1 **GGAAG**CGTTGTTACC**AGGTC**

ASO2 **TAGAG**TGAAATAGCG**GGTTC**

ASO3 **GTCCT**GGTTTCCTGT**TTTGT**

ASO4 **TGTTC**CTTGAAAAGT**GCTGG**

ASO5 **GGTTG**GAGTATGATT**AATGG**

#### Crosslinking-immunoprecipitation experiments

Crosslinking was performed in the same way as for the initial steps of iCLIP as previously described above ^9^. Lysates generated from the crosslinked cells were treated with 2 μl Turbo DNase and RNase I (1:250) for 3 min at 37°C. RNA-protein complexes were immunoprecipitated using 2μg anti-m6A antibody or Rabbit IgG as well as 25μl protein A/G Dynabeads. Following high salt washes, proteins were digested with proteinase K and the immunoprecipitated RNA extracted with Trizol and submitted to qRT-PCR. Input RNA reverse transcribed into cDNA was used to calculate the percent enrichment in the immunoprecipitated samples.

#### In vitro m6A methylation and pull-down assay

*HOTTIP* RNA probe was generated by In vitro transcription using T7 polymerase (Thermo Fisher Scientific #EP0113) or HiScribe T7 high-yield RNA synthesis kit (New England Biolabs #E2040S) according to the manufacturer’s instructions. 0.15nmol of in vitro transcribed RNAs were denatured by heating at 90°C for 3 min and refolded, and were incubated in 1 × methyltransferase buffer (80 mM KCl, 1.5mM MgCl_2_, 0.2U/ul RNase inhibitor, 10 mM DTT, 4% glycerol and 15mM HEPES PH7.9), together with 0.8 mM SAM and purified METTL3/14 (with a molar ration 0.15nmol/0.15nmol) (+m6A) for 12 hours at 16°C. Control reactions contained no METTL3/14 (−m6A).Then the purified RNAs were biotinylated using RNA 3’ end desthiobiotinylation kit (Thermo Fisher Scientific) according to the manufacturer’s instructions. The RNA-protein pull down assay was performed using Pierce magnetic RNA-protein pull-down kit (Thermo Fisher Scientific Cat# 20164). The complexes were washed and analyzed by western blotting ^10^.

#### Purification of METTL3/14

HEK293T cells were transfected with plasmids expressing pcDNA3.1-FLAG-METTL3, pcDNA3.1-FLAG-METTL14 (Addgene Cat#53739 and 53740). Cell pellets were resuspended in 1× lysis buffer (10 mM HEPES (pH 7.6), 150 mM KCl, 2 mM EDTA, 0.5% NP-40, 0.5mM DTT, 1× protease inhibitors) and incubated for 10 minutes at 4°C. Cell lysate was clarified by centrifugation at 4°C, 12,000 × g for 15 minutes. Supernatant was transferred to a new tube and incubated with anti-Flag M2 beads in 1× lysis buffer (200mM NaCl, 50mM HEPES, 2mM EDTA and 0.05% NP-40, 0.5mM DTT, 1× protease inhibitors) for 2-4 hours at 4°C. For elution, supernatant was removed, and the beads was washed and incubated with 3×Flag peptide (Thermo Fisher F3290) for 2 hours at 4°C with rotation. The elutes were analyzed by Coomassie to determine protein concentration and purity. Samples were aliquoted and stored at −80°C and thawed on ice prior to use for in vitro m6A methylation experiments.

#### Chromatin immunoprecipitation (ChIP) assay

ChIP assay was carried out as described previously ^11^. Briefly, 5×10^6^ cells were crosslinked in 1% formaldehyde for 10 min at room temperature, and then quenched by 125 mM glycine for 5 min at room temperature, and washed with ice-cold 1x PBS twice. Cells were then lysed in ChIP lysis buffer (50 mM Tris-HCl, pH 8, 10 mM EDTA, 1% SDS, protease inhibitor cocktail). After that, chromatin DNA was sheared into fragments by sonication with the Bioruptor^™^ UCD200. Lysates were centrifuged at 14,000 rpm for 10 min at 4°C and diluted in ChIP buffer (20 mM Tris-HCl, pH 8.0, 2 mM EDTA, 1% Triton X-100, 150 mM NaCl), and 10% of the lysate was used as an input. Chromatin was immunoprecipitated with indicated and IgG antibody overnight at 4°C. Then, each sample was incubated with 50 μL Protein G Dynabeads^™^ (Thermo Fisher Scientific) for 2 hrs at 4°C and washed sequentially with low salt washing buffer (20 mM Tris-HCl, pH 8.0, 150 mM NaCl, 2 mM EDTA, 1% Triton X-100, 0.1% SDS), high salt washing buffer (20 mM Tris-HCl, pH 8.0, 500 mM NaCl, 2 mM EDTA, 1% Triton X-100, 0.1% SDS), lithium chloride washing buffer (10 mM Tris-HCl, pH 8.0, 1 mM EDTA, 1% Triton X-100, 250 mM LiCl, 1% sodium deoxycholate), and TE buffer (50 mM Tris-HCl, pH 8.0, 10 mM EDTA). Chromatin DNA was eluted for 30 min at room temperature in elution buffer (100 mM NaHCO_3_, 1% SDS), reverse crosslinked with 2 μl of 10 mg/mL proteinase K overnight at 65°C. After that, chromatin DNA was purified, and analyzed by quantitative PCR. Results represent percentage of input and error bars indicate standard deviations (S.D.) through triplicate experiments.

ChIP-DNA libraries were prepared with NEBNext Ultra II DNA Library Prep Kit for Illumina (E7645S, New England Biolabs). Briefly, 10 ng ChIP DNA fragments were used to perform end repair and then purified with AMPure XP beads. After that, the 3’ end adenylation was performed. Then, fragments were ligated with adapter indexes and amplified with adapter primers. Quality control was performed with Qubit and Agilent Bioanalyzer and libraries were subjected to paired-end sequencing at 150 bp length on an Illumina NovaSeq 6000 platform.

#### Chromatin Isolation by RNA Immunoprecipitation (ChIRP) assay

The Chromatin Isolation by RNA Immunoprecipitation (ChIRP) assay was performed as described previously ^12^ with some modifications. In brief, 20 million cells were cross-linked with 1% glutaraldehyde for 10 min at room temperature with shaking, and then quenched by 125 mM glycine at room temperature for 5 min, washed with ice-cold PBS twice. Cells were then lysed in 1mL of cell lysis buffer (50 mM Tris-HCl pH 7.0, 10 mM EDTA, 1% SDS, add PMSF, DTT, P.I. and Superase-in in fresh), and then chromatin DNA was sheared into fragments by sonication with a Bioruptor^™^ UCD200 (Diagenode). Chromatin was diluted in hybridization buffer (750 mM NaCl, 1% SDS, 50 mM Tris-HCl pH 7.0, 1 mM EDTA, 15% formamide, add DTT, PMSF, P.I, and Superase-in fresh) and hybridized with 100 pmole of biotinylated DNA probes targeting *HOTTIP,* and LacZ probe as a negative control. DNA/RNA hybrids were precipitated using 100μL of Streptavidin-magnetic C1 beads (Invitrogen) and washed 5 times with washing buffer (2x SSC, 0.5% SDS). RNA was isolated using TRIzol reagent and *HOTTIP* enrichment was confirmed by RT-qPCR, with *β-actin* as a negative control. DNA was isolated using Phenol:Chloroform extraction and ethanol precipitation. ChIRP-seq library was prepared for sequencing as described for ChIP-seq library.

ChIRP-WB or ChIRP-mass spectrometry (ChIRP-MS) were carried out as previous described ^13^. Briefly, total proteins were eluted from the beads by shaking for 20 min at room temperature followed by incubation at 65 °C for 10 min. Then, proteins were precipitated by adding 50 μL TCA (25% v/v) and vortexed overnight at 4 °C. Proteins pellet were spun down at 16,000 g at 4 °C for 30 min, washed once with cold acetone, and then air-dried for 1 min, and solubilized in 1× laemmli sample buffer. Proteins were reverse cross-linked at 95 °C for 30 min with occasional mixing and resolved on bis-tris SDS-PAGE gels for western blot, using the indicated antibodies or mass-spectrometry. ChIRP-MS (LC/MS/MS), experiments were carried out at Taplin Mass Spectrometry Facility at Harvard Medical School ^14-16^. Briefly, Coomassie brilliant blue gel bands were cut into approximately 1 mm^3^ pieces and subjected to a modified in-gel trypsin digestion procedure. Gel pieces were washed and dehydrated with acetonitrile for 10 min, the acetonitrile was removed, and they were completely dried in a SpeedVac Vacuum. Gel pieces were rehydrated with 50 mM ammonium bicarbonate solution containing 12.5 ng/μl modified sequencing-grade trypsin (Promega) for 45 min at 4°C. Subsequently, excess trypsin solution was removed and replaced with 50 mM ammonium bicarbonate solution to cover the gel pieces overnight at 37°C. Peptides were then extracted by removing the ammonium bicarbonate solution, washing once with a 50% acetonitrile/1% formic acid solution, and drying in a speed-vac. Next, peptides were subjected to electrospray ionization and entered in an LTQ Orbitrap Velos Pro ion-trap mass spectrometer (Thermo Fisher Scientific). Peptides were detected, isolated, and fragmented to produce a tandem mass spectrum of specific fragment ions for each peptide. Peptide sequences were determined by matching protein databases. Overrepresentation analysis of protein classes (protein enrichment) was performed using DAVID database (https://david.ncifcrf.gov, Version 6.8) ^5, 17^. Protein classes overrepresented in the *HOTTIP*-ChIRP were ranked according to their Benjamini-Hochberg-corrected *p*-values (*p*-value threshold 0.05).

#### DNA:RNA immunoprecipitation (DRIP) assay

DNA:RNA immunoprecipitation (DRIP) assay was carried out as described previously ^18, 19^. Briefly, Ctrl, dALK-G2 and dALK-G3 MOLM13 AML cells were crosslinked in 1% formaldehyde for 10 min at room temperature, quenched by 125 mM glycine for 5 min at room temperature, and washed with ice-cold 1x PBS twice. Cells were then lysed in lysis buffer (50 mM HEPES-KOH pH 7.5, 140 mM NaCl, 1 mM EDTA pH 8.0, 1% Triton X-100, 0.1% Na-Deoxycholate, 1% SDS) and fragmented by sonication (Bioruptor) to 300bp. The fragmented chromatin was treated with RNase A (10 mg/mL) in TE buffer (10 mM Tris-HCl pH 8, 10 mM EDTA pH 8) at 37°C for 1 hr, followed by Proteinase K (10 mg/mL) at 65°C overnight. Nucleic acids were extracted by phenol-chloroform extraction, ethanol precipitation, and resuspension in 5 mM Tris-HCl pH 8.5. Two percent of each sample was retained for input DNA. Half of the samples were treated with RNaseH at 37°C overnight. 50 μL of BSA-blocked Protein A Dynabeads (Thermo Fisher Scientific) were incubated with 10 μg of S9.6 antibody in IP buffer (50 mM Hepes/KOH at pH 7.5; 0.14 M NaCl; 5 mM EDTA; 1% Triton X-100; 0.1% Na-Deoxycholate, ddH_2_O) at 4°C for 4 h with rotation. Purified genomic DNA was added and rotated at 4°C overnight. Beads were washed sequentially with low salt buffer (50 mM Hepes/KOH pH 7.5, 0.14 M NaCl, 5 mM EDTA pH 8, 1% Triton X-100, 0.1% Na-Deoxycholate), high salt buffer (50 mM Hepes/KOH pH 7.5, 0.5 M NaCl, 5 mM EDTA pH 8, 1% Triton X-100, 0.1% Na-Deoxycholate), LiCl wash buffer (10 mM Tris-HCl pH 8, 0.25 M LiCl, 0.5% NP-40, 0.5% Na-Deoxycholate, 1 mM EDTA pH 8), and TE (100 mM Tris-HCl pH 8, 10 mM EDTA pH 8) twice. Complexes were eluted in 100 μL of elution buffer (50 mM Tris-HCl pH 8, 10 mM EDTA, 1% SDS) for 15 min at 65°C, treated with RNaseH at 37°C for 30 min, and analyzed by qPCR.

#### Sonication DNA:RNA immunoprecipitation coupled to high throughput sequencing (sDRIP-seq)

sDRIP-seq was performed as previously described ^20^. Briefly, DNA from 5-6 millions of Ctrl and dALK MOLM13 AML cells were extracted and fragmented by sonication to 300-500bp. 10 μg of sonicated DNA were treated with 4 μL of RNase H for 1 to 2 hours at 37°C as a negative control. 4.4 μg of fragmented DNA in a final volume of 500 μL of TE buffer per tube, and 50 μL (1/10 of the volume) from each tube was used as an input. Then 50 μL of 10X binding buffer (100 mM NaPO4 pH 7, 1.4 M NaCl, 0.5% Triton X-100) and 5-10 μL of S9.6 antibody (1 mg/ml) was added to the 450 μL of diluted DNA and incubated overnight at 4°C. 50 μL of BSA-blocked Protein A Dynabeads (Thermo Fisher Scientific) were incubated at 4°C for additional 2 hours with rotation. After washing with the 1X binding buffer, 250 μL of elution buffer (50 mM Tris-Cl pH 8, 10 mM EDTA pH8, 0.5% SDS) and 7 μL of proteinase K (20 mg/mL stock) were added to the beads and incubate with rotation at 55°C for 45 minutes. Then the immunoprecipitated DNA was purified with Phenol/Chloroform Isoamyl alcohol (25:24:1) and ethanol precipitated, and the resuspended DNA was subjected to qPCR for DRIP efficiency check. For library preparation, DNA fragments were subjected to second strand synthesis, end repair (NEBNext End Repair Module E6050S), A-tailing by adding adenosine to the 3’ ends of fragment using Klenow fragment (New England Biolabs), and adaptors were ligated using NEB quick ligase (New England Biolabs #M2200S). AmpErase Uracil N-glycosylase (Thermo Fisher Scientific # N8080096) was added for a strand-specific sDRIP library production. To determine the optimal PCR cycles for optimal library preparation, the 30 cycles of qPCR reaction were performed with NEBNext^®^ Multiplex Oligos for Illumina (New England Biolabs #E7335S, E7500S). The optimal number of cycles was determined for each library by setting a threshold just before reaching the end of exponential amplification step. Final DNA libraries were selected using AMPure XP beads, quantified and analyzed using Qubit dsDNA HS Assay Kit (Thermo Fisher Scientific #Q32854). Illumina libraries were sequenced in paired-end at 150 bp length on Illumina NovaSeq 6000 platform.

#### ChIP-seq, sDRIP-seq and ChlRP-seq analysis

Briefly, after quality control by FastQC ^21^, the paired-end sequence reads were trimmed and filtered using cutadapt (http://cutadapt.readthedocs.io, version 1.18) ^1^. Then trimmed reads were mapped to the human (hg19) reference genome using Bowtie2 with default parameters ^22^. SAM files were converted to BAM files and sorted using Samtools ^23^. Peak calling was performed using MACS2 ^24^. A bigWig file, including fragment or read coverages for control and experimental datasets, was generated with the bedGraphToBigWig program (https://www.encodeproject.org/software/bedgraphtobigwig/). Sequencing tracks were viewed using the Integrated Genomic Viewer (IGV) ^25^. Peak annotation was performed by ChIPseeker (version 1.28.3). *de novo* motif analysis was performed using HOMER (v4.10) ^26^. Additionally, the heatmap and curve of the peak region distribution on the genome (including promoter and enhancer) were performed with computeMatrix and plotHeatmap program on deepTools software (v3.5.4) (https://deeptools.readthedocs.io/en/develop/index.html). Ontology analysis was performed using DAVID (https://david.ncifcrf.gov, Version 6.8) ^5, 17^. Differential peaks analysis was performed using the DiffBind (v3.12.0) packages (Cut-off: ≥1.5 fold change; P value ≤ 0.05) in R ^27^. Overlapping peak analysis was carried out using the “bedtools intersect” program from bedtools (v2.29.2)^28^. All datasets were deposited in the NCBI GEO under accession number (GSE310379, GSE114981, GSE165049).

#### HiC assay and sequencing library preparation

HiC assay was performed in dALK-Ctrl, dALK-G2 and dALK-G3 cells as previously described^11^. Arima HiC_+_ kit (A410030, Arima Genomics) was used for HiC-seq library preparation. 10M cells were cross-linked in 1 % formaldehyde (BP531, Fisher Bioregents) at room temperature for 10 min and stopped by 125 mM glycine solution. 1M of crosslinked cells were lysed and DNA yield per 1M cells was estimated. Approximately 3ug of total DNA was used for input. The cross-linked cell pellet was treated with lysis buffer and incubate at 4°C for 15 min. The extracted DNA from lysed cells was digested with restriction enzyme cocktail including in Arima-HiC Kit at 37°C for 30 min followed by at 65°C for 20 min. After the 5’-overhangs of the digested DNA was filled ends and labeled with a biotinylated nucleotide, digested end of DNA was treated with ligase and incubated at 25°C for 15 min. The ligated DNA was purified with DNA purification beads (AMPure XP Beads, Beckman Coulter) and the DNA concentration was measured by Qubit (Thermo Fisher Scientific).

1μg of purified DNA per sample was sheared by sonication (Bioruptor, Diagenode) with default parameters (30 seconds ON, 30 seconds OFF pulse intervals) to obtain average 400bp length. Fragmented DNA was size-selected to have a size distribution between 200-600 bp. After enrichment of biotinylated DNA, library preparation was performed with KAPA Hyper Prep Kit (KK8504, Roche). Final libraries were checked by BioAnalyzer (Agilent) and submitted to paired-end sequencing of 100 bp length on an Illumina Novaseq 6000 platform.

#### Hi-C sequencing Analysis

Raw sequencing reads were cleaned to remove adaptor sequence with cutadapt (ver.1.18) and trimmed the end of enzymatic sequences with homerTools (homerTools trim -3 GATC -mis 0 -matchStart 20 -min 20) from Homer software ver.4.10 ^26^. Trimmed reads were aligned to human reference genome (hg19) using Bowtie2 ver.2.3.4.3 ^22^ with parameters “--local --very-sensitive -p 20 -n 1”. Aligned sam files were used to make a tag directory by makeTagDirectory package from Homer software with “-tbp 1” parameter to remove PCR duplicates. After filtering uninformative reads using makeTagDirectory package with different parameters “-genome mm10 -removePEbg -restrictionSite GATC -both -removeSelfLigation -removeSpikes 10000 5”, the filtered tag directory file was converted to .hic files using tagDir2hicFile.pl. These interaction matrices for Hi-C heatmap were visualized by Juicebox ver.2.13.07 ^29^ using KR normalization with “Balanced” option.

#### Chromosome conformation capture combined with chromatin immunoprecipitation (HiChIP) and sequencing assay

HiChIP assay was performed in Ctrl, dALK-G3 MOLM13 AML cells, dALK-G3 vector control and dALK-G3 RAD21 as previously described with a minor modification ^30^. Briefly, 2 x 10^7^ cells were crosslinked with 1% formaldehyde for 10 min and subsequently quenched with 0.125M glycine. Chromatin was digested using DpnII restriction enzyme (NEB), followed by biotin incorporation with Biotin-14-dATP (Invitrogen). DNA was then ligated with T4 ligase (NEB) and sonicated on Bioruptor (10 cycles (30-sec on, 30-sec off) set at position “high”). Sheared chromatin was diluted with ChIP dilution buffer (15mM Tris pH 7.5, 150mM NaCl, 1.2mM EDTA, 0.01% SDS, 1.1% Triton X-100), and cleared with IgG (CST) and then incubated with anti-CTCF antibody (CST) at 4°C overnight. Chromatin-antibody complexes were captured by Protein-A magnetic beads at 4°C for 2 hrs, and subsequently washed with Low Salt Wash Buffer, High Salt Wash Buffer, LiCl Wash Buffer and eluted with elution buffer. After that, chromatin DNA was purified with MinElute PCR Purification Kit (Qiagen) and quantified using Qubit High Sensitivity Assay kit (Thermo Fisher Scientific). 50-150ng was used for capture with Dynabeads MyOne Streptavidin C-1 (Thermo Fisher Scientific) and 10 ng of chromatin with 0.5 μL of Tn5 enzyme (Illumina) was added to captured DNA to generate sequencing library. A first PCR in a final volume of 50μl with 5 cycles was performed (72°C for 5 min, 98°C for 1 min, then 5 cycles at 98°C for 15 s, 63°C for 30 s, and 72°C for 1 min). To determine the additional PCR cycles for optimal library preparation, the 30 cycles of qPCR reaction was performed with 5 μL of the first PCR product, 1 μL of Nextera Ad1_noMX (universal primer) and Nextera Ad2.X (each at 1.25 μM) primers, 5 μl of phusion HF 2X (New England Biolabs), 0.75 μL Evagreen^®^ PCR MIX (Biotium) and 2.25 μL of water. The optimal number of additional cycles was determined for each library by setting a threshold just before reaching the end of exponential amplification step. The 45μl left from the first PCR were further amplified using additional PCR c ycles as determined above. Around 300 bp DNA fragments was selected using Ampure XP beads. Libraries were quantified and analyzed using Qubit dsDNA HS Assay Kit (Thermo Fisher Scientific). Illumina libraries were sequenced paired-end at 150 bp length on Illumina NovaSeq 6000 platform.

#### HiChIP sequencing data analysis

HiChIP-seq data analysis was carried out with the standard procedure according to the manufacturer’s instruction (Arima Genomics)^30^. Paired-end raw fastq reads from HiChIP experiments were trimmed and aligned to the human hg19 reference genome using HiC-Pro ^31^. After PCR duplicates and low-quality reads were removed, high-quality reads were assigned to DpnII restriction fragments, and then filtered for valid interaction paired-end tags (PETs). Then, these filtered reads were used to generate a 5kb interaction matrix of valid pairs ^31^. For further analysis of HiChIP contact maps, HiC-Pro was used to generate “.hic” file for data visualization in Juicebox software ^29^. HICCUPS loop-calling tool (juicer_tools_1.22.01.jar hiccups --cpu -m 500 -r 5000,10,000) was performed to identify chromatin loops ^32^. FitHiChIP (https://ay-lab.github.io/FitHiChIP/) ^33^ was performed to call significant interactions with default parameters with FDR cutoff at 0.05 for finding long-range contacts associated with CTCF proteins. Further analysis of long range chromatin DNA differential loops (gains and losses of loops) were identified the significant variations in topology between Ctrl and dALK-G3 samples with diffloop program ^34^. Finally, the WashU Epigenome Browser was used to explore and visualize the variations in chromatin interactions between samples ^35^.

#### m6A eCLIP (enhanced crosslinking immunoprecipitation) sequencing assay

m6A eCLIP (MeCLIP) assay was performed in Ctrl, dALK-G2 and dALK-G3 MOLM13 AML cells, as previously described with modifications ^36, 37^. Briefly, total RNA was purified from MOLM13 cells and treated with DNase, and poly(A)+ RNA was prepared by depleting ribosomal RNA using Ribominus (Thermo Fisher Scientific K155002). 5-20 μg of mRNA were incubated with RNA Fragmentation Reagents (Invitrogen AM8740) to fragment the RNA. The mixture was incubated in a heat block at 70°C for 5-15 minutes to achieve RNA fragment 30-175bp, after which the stop solution (Invitrogen AM8740) was added to the reaction. The fragmented RNA was diluted in 450 μl dilution buffer (50 mM Tris, pH 7.4, 100 mM NaCl, 0.05% NP-40) and incubated with 1–5 μg anti-m6A at 4 °C for 1–2 hours. RNA-antibody complexes were UV crosslinked twice with 0.15 J/cm2 at 254 nm by a UV crosslinker (Fisher). Protein A/G Dynabeads (30 μL) were added, and the samples were rotated for 2 hours at 4°C, and were washed twice with high-salt buffer (50 mM Tris, pH 7.4, 1 M NaCl, 1 mM EDTA, 1% NP-40, 0.1% SDS), twice with dilution buffer, and twice with polynucleotide kinase (PNK) wash buffer (20 mM Tris, 10 mM MgCl2, 0.2% Tween 20). After dephosphorylation with FastAP (ThermoFisher) and T4 PNK (NEB), a barcoded RNA adapter was ligated to the 3' end (T4 RNA Ligase, NEB). Subsequently, SDS-PAGE electrophoresis and nitrocellulose membrane transfer was carried out, and then the 20-175 kDa region size-selected was performed by cutting from the membrane corresponding to RNA fragments crosslinked to antibody heavy and light chains. After that, RNA library preparation was performed as previously described with standard eCLIP ^37^, and input RNA was prepared in parallel. The final library’s yield was assessed by Agilent TapeStation. Quality control was carried out using Qubit and Agilent Bioanalyzer. MeCLIP-seq libraries were subjected to paired-end sequencing at 150 bp length on an Illumina NovaSeq 6000 platform.

#### MeCLIP-seq data analysis pipeline

MeCLIP-seq data analysis was performed as previously described ^37^. Briefly, the reads are initially inspected for appropriate quality using FastQC, and the in-line unique molecular identifier (UMI) was extracted using UMI-tools (v. 1.0.0). The remaining non-random DNA adapter and indexed RNA adapters are then removed using Cutadapt (version 1.2.0) with any reads less than 18bp being discarded. The trimmed reads are then briefly analyzed visually once more with FastQC to ensure all adapters are successfully removed. The reads were mapped with splicing-aware aligner HISAT2 (Version 2.2.1) or STAR (v 2.4.0i). To prevent false assignments of reads from repetitive regions, reads with a mapping quality < 5 were removed from further analysis. Subsequent removal of PCR duplicates was performed with UMI-tools. Peaks calling was identified within MeCLIP samples with the corresponding input samples as controls, using the peak caller MACS2, or PureCLIP ^38^ to find exact crosslink sites. PureCLIP calls individual crosslink sites from CLIP-seq data using a hidden Markov model approach. To identify crosslink sites, PureCLIP peaks were called on CLIP-seq data using input data as background signal. The identified significant peaks were utilized to extract underlying reads from each dataset and the resulting eCLIP reads were normalized to input reads (MeCLIP/input) using deepTools software (v3.5.4) (https://deeptools.readthedocs.io/en/develop/index.html). Overlapping peak analysis was carried out using the “bedtools intersect” program from bedtools (v2.29.2). The dataset was deposited in the NCBI GEO under accession number (GSE310379).

#### Xenotransplantation of human leukemic cells and Patient-Derived Xenografts (PDX)

All animal breeding and experiments were approved by and conducted in accordance with the IACUC of the University of Texas Health Science Center at San Antonio and the Pennsylvania State University College of Medicine. Adult NOD.*Cg-Prkdc*^scid^*Il2rg*^tm1Wjl^/SzJ (NSG) mice (6-8 weeks old) were pretreated with 280cGy total body irradiation or 2 dose of busulfan (20 mg/kg/day), then 2x10^6^ cells of Ctrl and dALK-G3 MOLM13 or 1.8 × 10^5^ primary patient AML cells were transplanted into each NSG mice by tail-vein injection. At 18-24 days after transplantation, peripheral blood was collected and depleted of red blood cells by ammonium chloride treatment, BM was isolated from the femurs, and the spleen was processed into a single cell suspension. Human CD45^+^ chimerism in the BM, spleen, and PB cells were analyzed by flow cytometry (FACS LSR II—BD Biosciences, San Jose, CA, USA).

### QUANTIFICATION AND STATISTICAL ANALYSIS

Statistical differences were determined by Student's t-test or analysis of variance (ANOVA) followed by Newman-Keuls multiple comparison tests. * indicates p-value less than 0.05, ** indicates p-value less than 0.01, and *** indicates p-value less than 0.001. For *in vitro* experiments, at least three independent experiments with at least three biological replicates for each condition/genotype were carried out. For *in vivo* experiments, the sample size chosen was based on the generalized linear model with Bonferroni multiple comparison adjustments, the proposed sample size of at least five mice/group/genotype according to that, and animals were randomly assigned to each study.

## Supplementary Material

1

This is a list of supplementary files associated with this preprint. Click to download.

- SupplementaryTableS1ver1.xlsx

## Figures and Tables

**Figure 1.**
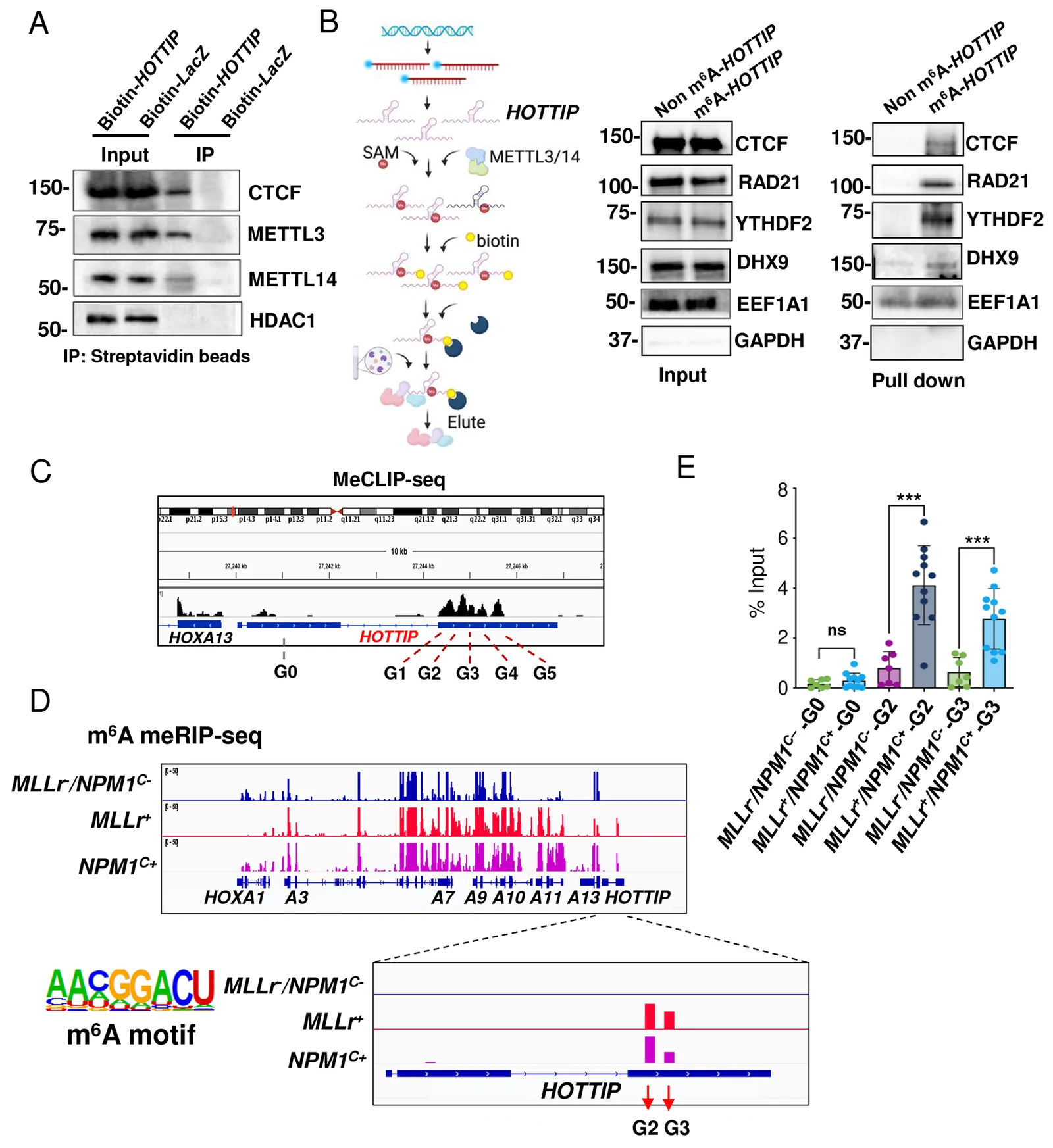
*HOTTIP* associates with m^6^A modifiers/readers in highly enriched in m^6^A modification in AML patients. **(A)** ChIRP western blots showing the interactions between *HOTTIP* and CTCF or m^6^A modifiers, METTL3/METTL14. MOLM13 cell lysate was incubated with biotin labeled probes targeting *HOTTIP* or *lacZ* and the associated proteins were subjected to western blot and probed with indicated antibodies. **(B)** *In vitro* methylation and pull-down assay showing m^6^A methylated *HOTTIP* interaction with CTCF/cohesin, R-loop regulator, DHX9, and specific m^6^A reader, YTHDF2. *HOTTIP* RNA was synthesized *in vitro*, and the methylation assay was carried out with or without adding purified METTL3/14 complex. The biotinylated RNA probes were incubated with nuclear extracts from MOLM13 cells, and the bound proteins were subjected to western blot and probed with indicated antibodies. **(C)** Snapshots of m^6^A-eCLIP (MeCLIP) -seq at the *HOTTIP* locus in MOLM13 cells. MeCLIP-seq was carried out using UV crosslinking of the anti-m^6^A antibody to the fragmented mRNA. Arrows indicate the m^6^A sites (G1-G5) on the exon 3 of *HOTTIP,* and a non-m^6^A site region (G0) in exon 2. **(D)** m^6^A RIP-seq comparing among primary AML patients with or without *MLLr*^+^/*NPM1*^*C+*^ mutation. *Top,* Snapshots of m^6^A RIP-seq signal at the *HOXA* and *HOTTIP* locus. RIP experiments were performed using anti-m^6^A antibody in one *MLLr*^+^ AML patient, one *NPM1*^C+^ AML patient sample, and one *MLLr*^−^/*NPM1*^C−^ AML patient cells. *Bottom Left,* m^6^A-RIP identified the consensus motif enriched in patients harboring *MLLr*^+^/*NPM1*^*C+*^ mutations. *Bottom Right,* zoomed snapshots of m^6^A RIP-seq signal specifically only enriched on the G2 and G3 m^6^A sites *in HOTTIP* locus. **(E)** m^6^A RIP-qPCR comparing AML patients with or without *MLLr*^+^*/NPM1*^C+^ mutation. RIP experiments were performed using anti-m^6^A antibody in *MLLr*^+^*/NPM1*^*C+*^ AML patients (n=11) and *MLLr*^−^*/NPM1*^C−^ AML patient cells (n=7). IgG was used as a negative control for RIP experiments. The bound RNA was analyzed by RT-qPCR with primers targeting different m^6^A sites (G2 and G3) on *HOTTIP*. Primers targeting a non-m^6^A site (G0) on the exon 2 is shown as a negative control. Error bars denote standard deviation (SD). *p < 0.05, **p < 0.01, ***p < 0.001, n.s., not significant by ANOVA following Bonferroni post hoc test.

**Figure 2.**
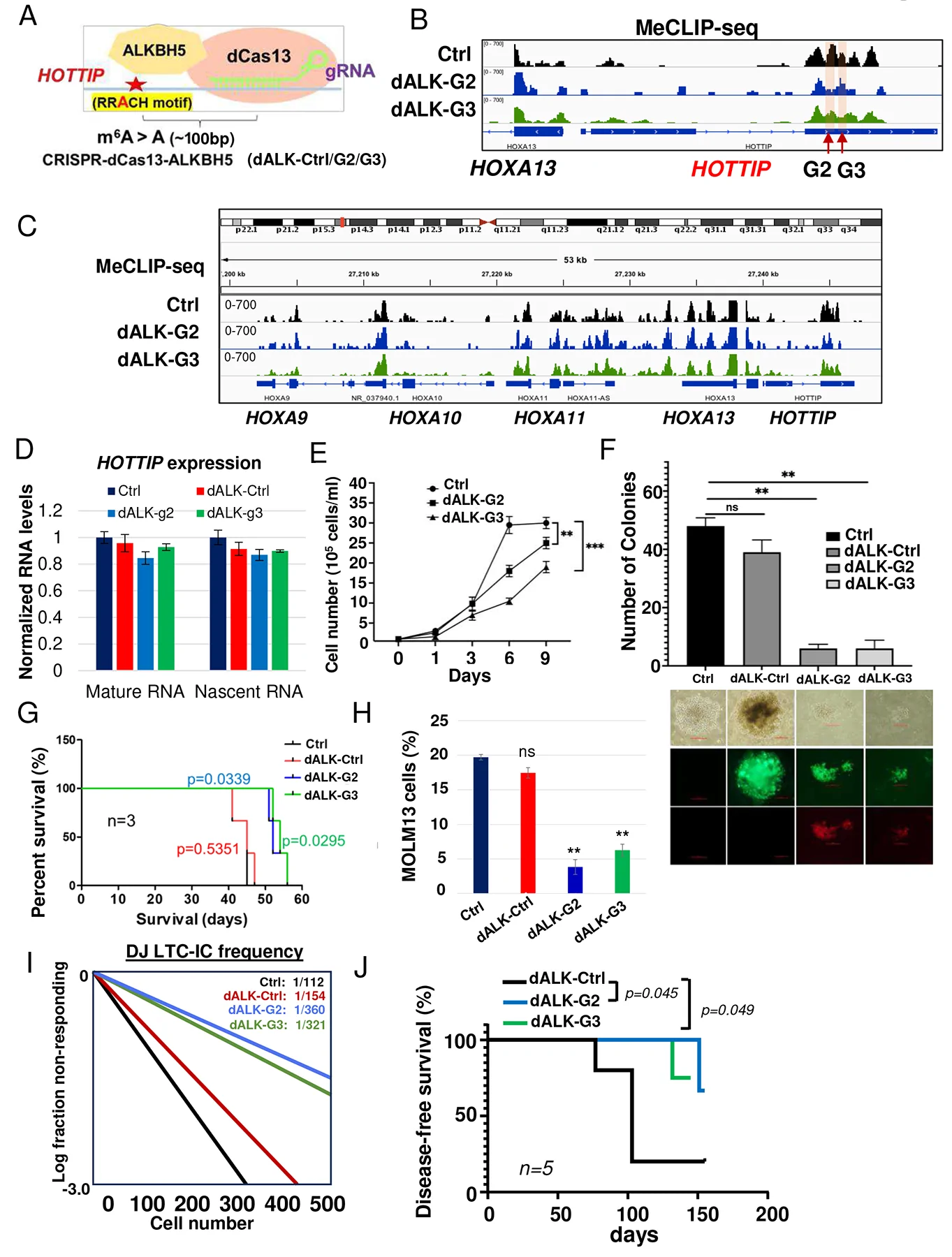
Erasing m^6^A modification at specific sites of *HOTTIP* RNA impairs *MLLr*^+^ AML LSC proliferation and prolongs survival of transplanted AML mouse models without affecting *HOTTIP* mRNA **(A)** Schematic representation of the constructed MOLM13 cells stably expressing dCas13-ALKBH5 and sgRNA plasmids targeting *HOTTIP* m^6^A sites for the constitutive demethylation at *HOTTIP* m^6^A sites. The star symbol indicates the RRACH motif, and the gRNA sequence are designed within 100 bp from the consensus m^6^A RRACH motif. **(B)** Snapshots of MeCLIP-seq at the *HOTTIP* locus. MeCLIP-seq was carried out in MOLM13 cells stably expressing control vector, or with sgRNAs targeting G2 m^6^A site (dALK-G2) or G3 m^6^A site (dALK-G3). **(C)** Snapshots of MeCLIP-seq at the whole *HOXA* loci. MeCLIP-seq was performed in MOLM13 cells stably expressing control vector, or dCas13-ALKBH5 targeting *HOTTIP* G2 or G3 m^6^A sites. **(D)** *HOTTIP* expression by RT-qPCR analysis. RNAs were isolated from MOLM13 cells stably expressing control vector, dCas13-Ctrl, or dCas13-ALKBH5 (G2 or G3) plasmids. The mature mRNA and nascent RNA expression levels of *HOTTIP* were normalized to *β-actin* mRNA. Error bars denote standard deviation (SD). **(E)** Effects of m^6^A of *HOTTIP* on cell proliferation. MOLM13 cells stably expressing control vector, dCas13-Ctrl, or dCas13-ALKBH5 (G2 or G3) constructs were seeded and counted at the indicated time points. Error bars denote SD. *p < 0.05, **p < 0.01, ***p < 0.001 (two-way ANOVA followed by post-hoc tests) **(F)** Changes in frequencies of colony formation comparing dALK control, dALK-G2, or dALK-G3 cells (dCas13-ALKBH5 vector is labeled with GFP; gRNA is labeled with RFP). Cells were cultured for 21 days and the colonies were counted under fluorescent microscopy. **(G)** Kaplan-Meier survival curves of NSG mice transplanted with dALK control, dALK-G2 or dALK-G3 MOLM13 cells. P-value was calculated by log-rank test followed by Bonferroni post hoc test. n=3 per group. **(H)** Changes in MOLM13 cell engraftment (hCD45+ cells) after transplantation with dALK control, dALK-G2 or dALK-G3 cells at day 40 post-transplantation. Percentage of MOLM13 cells in bone marrow from mice transplanted with indicated cells. Statistical significance was determined using one-way ANOVA with post hoc test. p < 0.05 (*), p < 0.01 (**), ns = not significant. **(I)** Long term culture-initiating cell (LTC-IC) frequency in AML patient cells expressing control or dCas13-ALKBH5 constructs (dALK-G2, dALK-G3). The frequency of LTC-ICs was calculated using limiting dilution analysis (LDA). **(J)** Kaplan-Meier survival curves of NSG mice transplanted with primary AML patient cells expressing dCas13-ALKBH5 (dALK) alone, or with sgRNAs targeting G2 m^6^A site (dALK-G2) and G3 m^6^A site (dALK-G3). P-value was calculated by log-rank test followed by Bonferroni post hoc test. n=5 per group.

**Figure 3.**
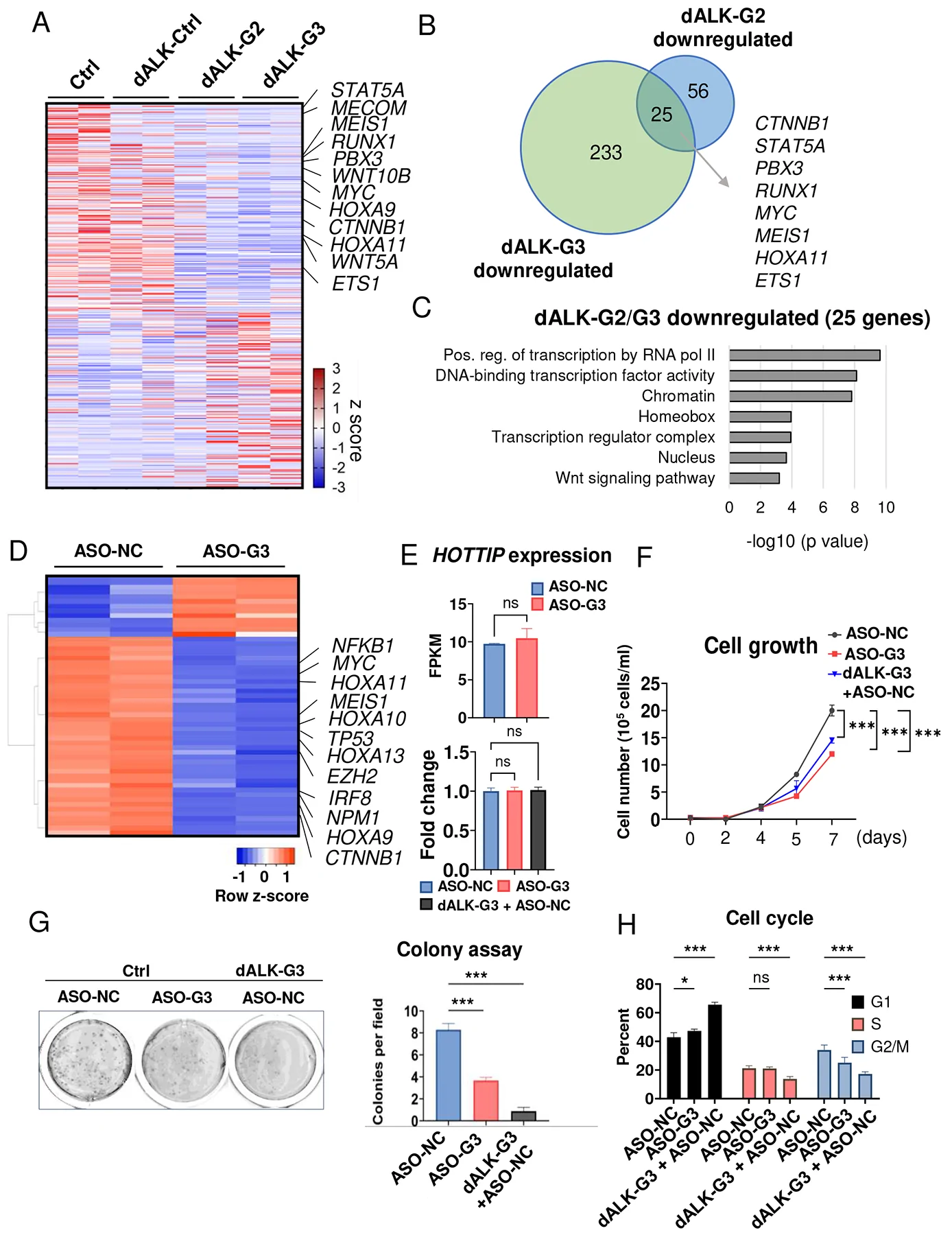
Erase of m^6^A modification at specific sites of *HOTTIP* impairs MLL target gene transcription and AML cell growth. **(A)** Heatmap of z-scores in differentially expressed genes (DEGs) upon G2 or G3 m^6^A modification erased on *HOTTIP* lncRNA by dCas13-ALKBH5. RNA-seq was performed on RNAs that were isolated from MOLM13 cells stably expressing control vector (Ctrl), dCas13-Ctrl, or dCas13-ALKBH5 targeting G2 or G3 sites. DEGs were selected by Cuffdiff using fold change (FC)>2 and p<0.01. **(B)** Venn diagram showing the overlapping of downregulated genes in dALK-G2 and in dALK-G3 MOLM13 cells identified by RNA-seq. **(C)** Gene ontology (GO) analysis of the overlapped 25 genes comparing dALK-G2 and dALK-G3 by RNA-seq. The downregulated genes were defined by Cuffdiff with a threshold of fold change (FC)>2, p<0.01. **(D)** Heatmap of z-scores in selected DEGs identified in RNA-seq analysis of ASO-mediated blockage of G3 m^6^A site on *HOTTIP* lncRNA. RNA-seq was performed on RNAs isolated from the MOLM13 cells that were treated with either steric-blocking ASO targeting G3 m^6^A site (ASO-G3) or a non-targeting control (ASO-NC). Representative DEGs were selected by Cuffdiff using FC>2 and FDR adjusted p value<0.05. Heatmap showing hierarchical clustering of genes (rows) and samples from biological duplicates (columns) using rank-correlation distance and average linkage. **(E)** *HOTTIP* expression is not affected by ASO-G3 blockage by RNA-seq and RT-qPCR analysis. Top, FPKM values from RNA-seq CuffDiff analysis. Bottom, RNAs were isolated from MOLM13 cells treated with either steric-blocking ASO targeting G3 m6A site (ASO-G3) or a non-targeting control (ASO-NC). dALK-G3 MOLM13 cells treated with ASO-NC serve as positive control. The mRNA expression levels of *HOTTIP* were normalized to *β-actin* mRNA. Error bars denote standard error of the mean (SEM). n.s., not significant (two-way ANOVA with post hoc test). **(F)** Effects of ASO targeting m^6^A of *HOTTIP* on cell proliferation. MOLM13 control cells or dCas13-ALKBH5 G3 cells were treated with either ASO targeting G3 m^6^A site or a non-targeting control, and the transfected cells were seeded and counted at the indicated time points. Error bars denote standard error of the mean (SEM). ***p < 0.001 (two-way ANOVA followed by post-hoc tests) **(G)** Changes in frequency of colony formation of m^6^A-targeting ASO-treated cells. MOLM13 control cells or dCas13-ALKBH5 G3 cells were treated with either steric-blocking ASO targeting G3 m^6^A site (ASO-G3) or a non-targeting control (NC), and the transfected cells were seeded in soft agar for 21 days and the colonies were stained by crystal violet. Error bars denote standard error of the mean (SEM). ***p < 0.001 (two-way ANOVA with post hoc test). **(H)** Cell cycle analysis of m^6^A-targeting ASO-treated cells. MOLM13 control cells or dCas13-ALKBH5 G3 cells were treated with either steric-blocking ASO targeting G3 m^6^A site or a non-targeting control, and the transfected cells were subjected to flow cytometry after 48 hours, and cell percentage in different cell cycle phases were quantified. Error bars denote standard error of the mean (SEM). *p < 0.05, **p < 0.01, ***p < 0.001. n.s., not significant (two-way ANOVA with post hoc test).

**Figure 4.**
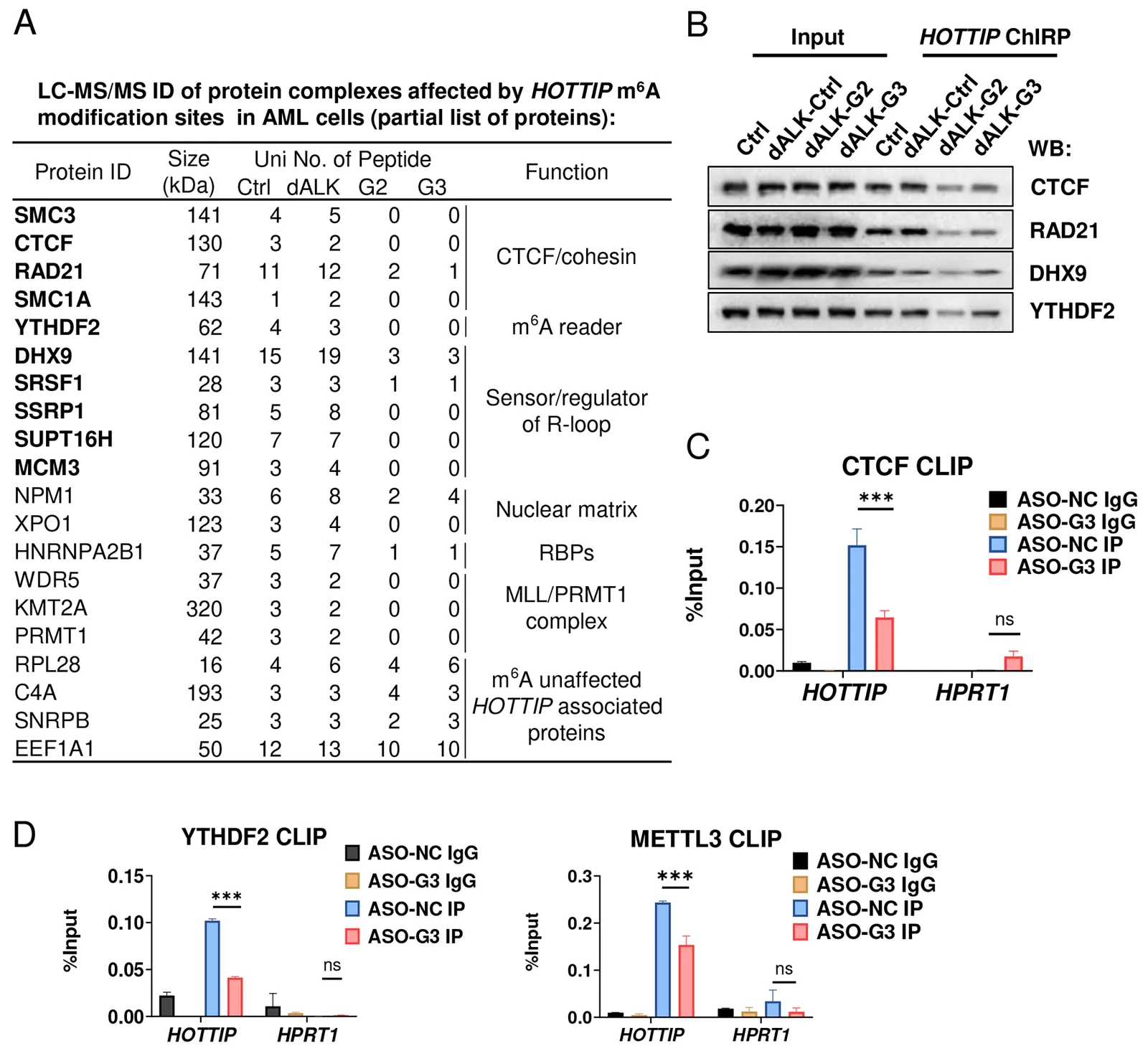
Erase of m^6^A modification at specific sites of *HOTTIP* disrupted *HOTTIP* interactions with CTCF/cohesin complex and R-loop sensors/regulators. **(A)** Partial list of unique peptides identified in *HOTTIP* ChIRP mass spectrometry comparing with or without specific erase of m^6^A modification on *HOTTIP*. *HOTTIP* ChIRP was performed in MOLM13 cells stably expressing control vector, dCas13-Ctrl, or dCas13-ALKBH5 targeting G2 or G3 site using biotinylated *HOTTIP* probes, and the eluted proteins were subjected to mass spectrometry analysis. Table includes annotated protein IDs, molecular weights, peptide numbers in each condition, and functional annotations. Protein classes overrepresented in the *HOTTIP*-ChIRP were ranked according to their Benjamini-Hochberg-corrected p-values (p-value threshold 0.05). **(B)** ChIRP western blots showing the changes in interactions between *HOTTIP* and downstream effectors upon removal of m^6^A modification on G2 or G3 site of *HOTTIP*. MOLM13 cell lysate expressing control vector, dCas13-Ctrl, or dCas13-ALKBH5 targeting G2 or G3 site was incubated with biotin labeled probes targeting *HOTTIP,* and the associated proteins were subjected to western blot and probed with indicated antibodies. **(C)** CTCF CLIP-qPCR to examine the changes in binding of CTCF to *HOTTIP* RNA upon ASO blockage of m^6^A modified G3 site on *HOTTIP*. CLIP experiments were performed using anti-CTCF antibody in MOLM13 cells that were treated either with ASO targeting G3 site or a non-targeting negative control (ASO-NC). IgG was used as a negative control for CLIP experiments. Error bars denote SEM. *p < 0.05, **p < 0.01, ***p < 0.001, n.s., not significant (two-way ANOVA with post hoc test). **(D)** YTHDF2 (Left) or METTL3 (Right) CLIP-qPCR to examine the changes in interaction between *HOTTIP* RNA and m^6^A reader YTHDF2 or writer protein METTL3 upon ASO blockage of m^6^A modified G3 site on *HOTTIP*. CLIP experiments were performed using anti-YTHDF2 or anti-METTL3 antibody in MOLM13 cells treated either with ASO targeting G3 site or non-targeting negative control (ASO-NC). IgG was used as a negative control for CLIP experiment. *HPRT1* was used as a non-enrichment RNA control. Error bars denote SEM. *p < 0.05, **p < 0.01, ***p < 0.001, n.s., not significant (two-way ANOVA with post hoc test).

**Figure 5.**
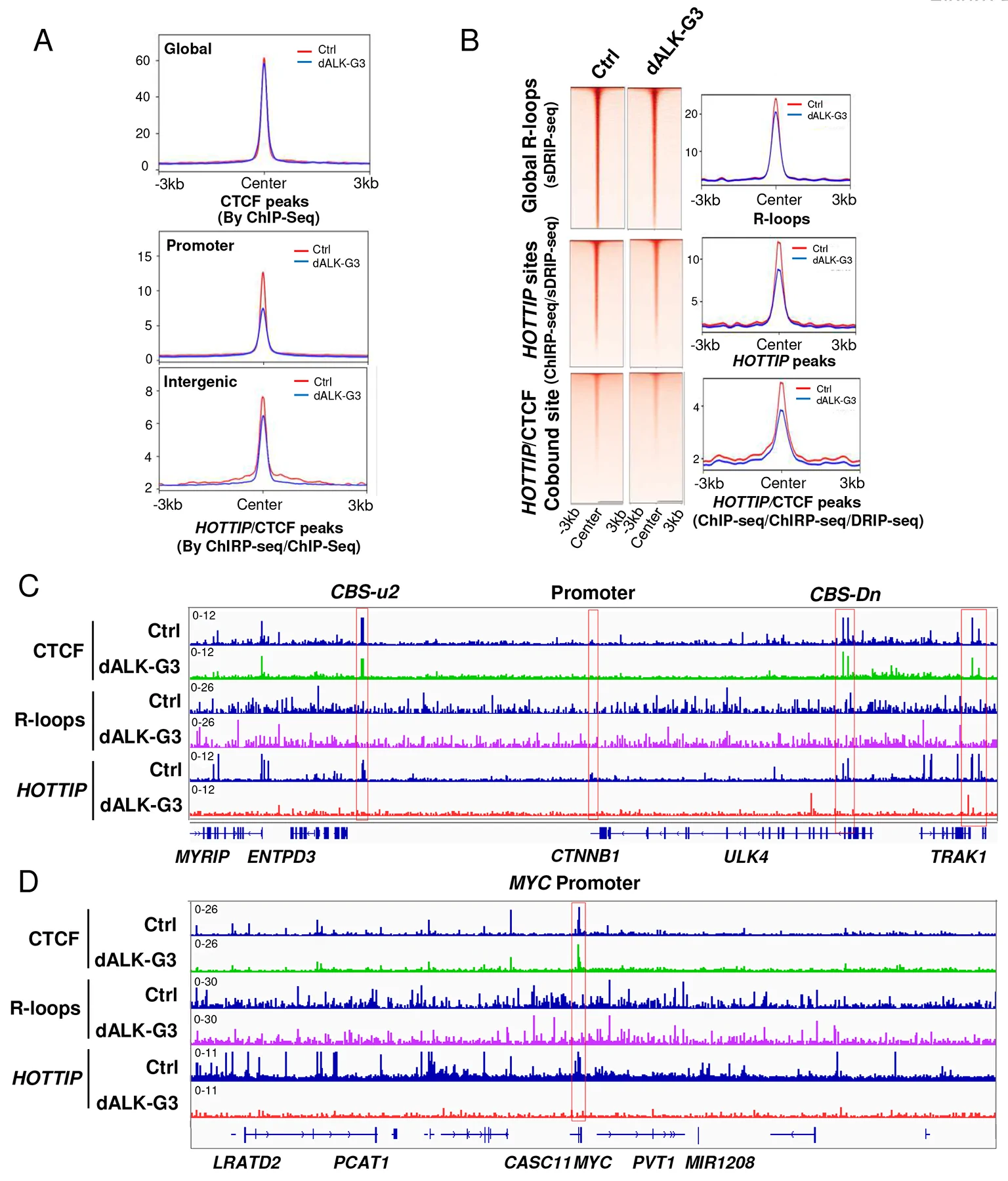
Site-specific m^6^A on *HOTTIP* regulates R-loop formation at *HOTTIP*/CTCF co-bounded sites **(A)** Plots of changes in global CTCF binding (Top) and at *HOTTIP/*CTCF co-binding sites (Bottom). CTCF ChIP-seq and *HOTTIP* ChIRP-seq were performed in Ctrl and dALK-G3 MOLM13 cells. Upper panel, metaplots represent the averaged global CTCF-ChIP signals in the genome-wide regions. Signals within ± 3000 bp of the peaks are shown. Middle and Lower panels, metaplots represent the averaged CTCF-ChIP signals in the co-bound promoter or intergenic regions. **(B)** Plots of changes in R-loop peaks from sDRIP-seq in genome-wide (Top) and at *HOTTIP* binding sites (Middle), and *HOTTIP*/CTCF co-bounded sites (Bottom). sDRIP-seq, CTCF ChIP-seq and *HOTTIP* ChIRP-seq were performed in Ctrl and dALK-G3 MOLM13 cells. Upper panels, heatmaps of sDRIP-seq in genome-wide scale (Left). Metaplots represent the averaged sDRIP-seq signals in the genome-wide regions (Right). Signals within ± 3000 bp of the peaks are shown. Middle panels, heatmaps of sDRIP-seq signals at *HOTTIP*-bound regions identified by *HOTTIP* ChIRP-seq (Left). Metaplots represent the averaged sDRIP-seq signals in the *HOTTIP*-bound regions (Right). Lower panels, heatmaps of sDRIP-seq signals at co-bound regions identified by CTCF ChIP-seq and *HOTTIP* ChIRP-seq (Left). Metaplots represent the averaged sDRIP-seq signals in the co-bound regions (Right). **(C and D)** Snapshots of CTCF binding, R-loops peaks and *HOTTIP* binding at *CTNNB1* (**C**) and *MYC* (**D**) loci compared Ctrl and dALK-G3 MOLM13 cells. Red box highlighted regions indicate promoters or CTCF binding sites at TAD boundaries of *CTNNB1* (**C**) or *MYC* locus (**D**).

**Figure 6.**
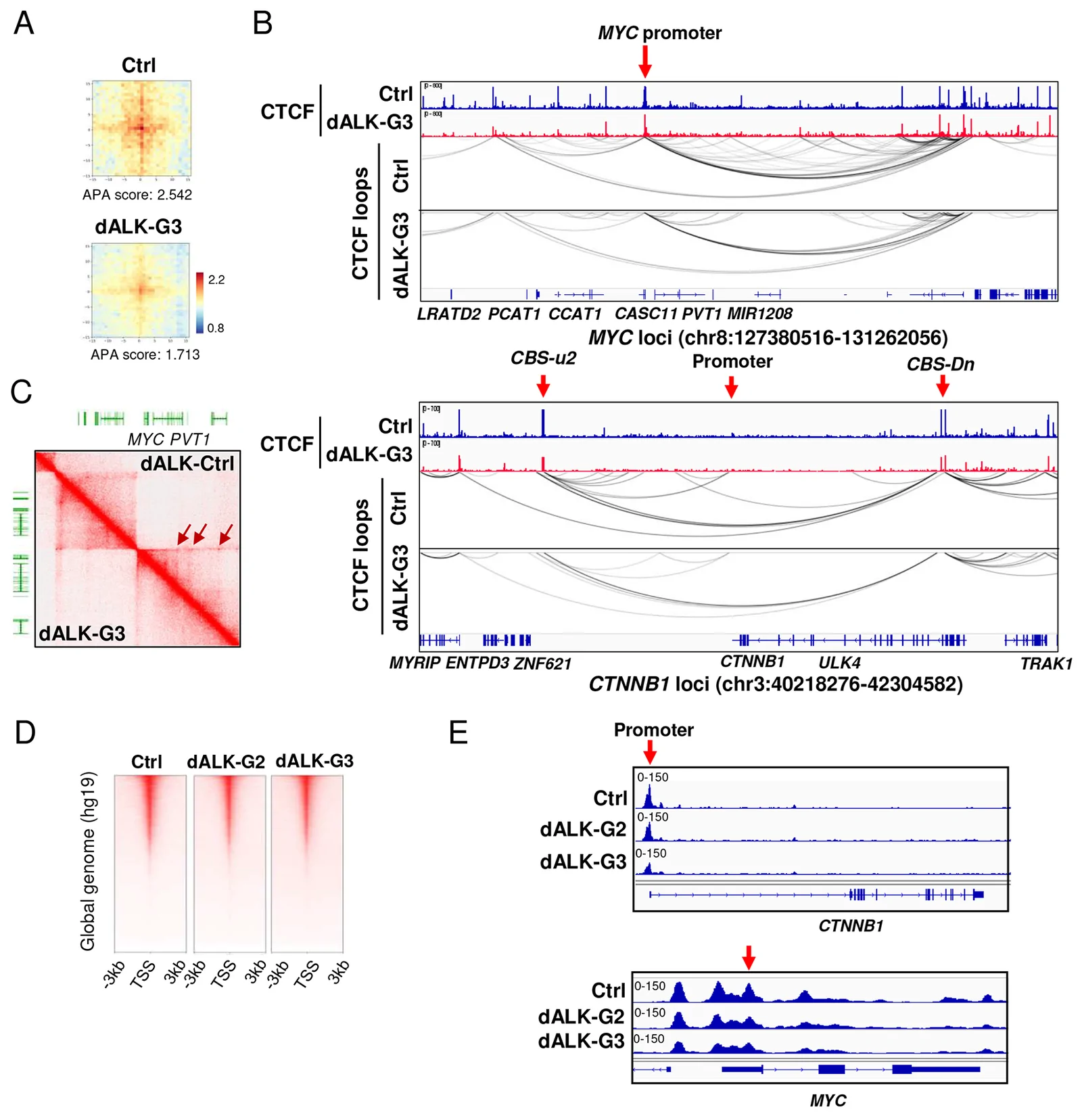
Site-specific m^6^A modification facilitates *HOTTIP*-dependent CTCF TAD integrity and CTCF-driven chromatin loops in AML genome. **(A)** APA (Aggregate Peak Analysis) plots of changed intensity of global CTCF-mediated chromatin loops comparing dALK-Ctrl and dALK-G3 cells by CTCF HiChIP. CTCF HiChIP were performed in Ctrl and dALK-G3 MOLM13 cells. APA scores are shown at the bottom of each plot. **(B)** Snapshots of CTCF binding and CTCF-mediated loops at *MYC* (Top) and *CTNNB1* (Bottom) loci comparing Ctrl and dALK-G3 MOLM13 cells, identified by CTCF HiChIP. Red arrows indicate CTCF-mediated loop anchors that are overlapped with CTCF binding sites at promoter and TAD boundaries of *MYC* and *CTNNB1* loci. **(C)** HiC-seq interaction tracks comparing dALK-Ctrl and dALK-G3 at *MYC* locus (Chr8:127600000-1297000000). Red arrows indicate reduced HiC signals upon erase of m^6^A modified G3 site on *HOTTIP*. HiC interaction matrix is normalized by the Knight-Ruiz (KR) matrix balancing. **(D)** Heatmaps of changes in global chromatin accessibility in genome-wide scale upon erase of m^6^A modified G2 or G3 site on *HOTTIP* detected by ATAC-seq. ATAC-seq was performed in Ctrl, dALK-G2 and dALK-G3 MOLM13 cells. Heatmaps of ATAC-seq signals comparing among Ctrl, dALK-G2, and dALK-G3 cells. Signals within ± 3000 bp of the TSS (transcription start site) are shown. **(E)** Snapshot of ATAC-seq tracks at *CTNNB1* and *MYC* loci comparing chromatin accessibility in Ctrl, dALK-G2 and dALK-G3 cells.

**Figure 7.**
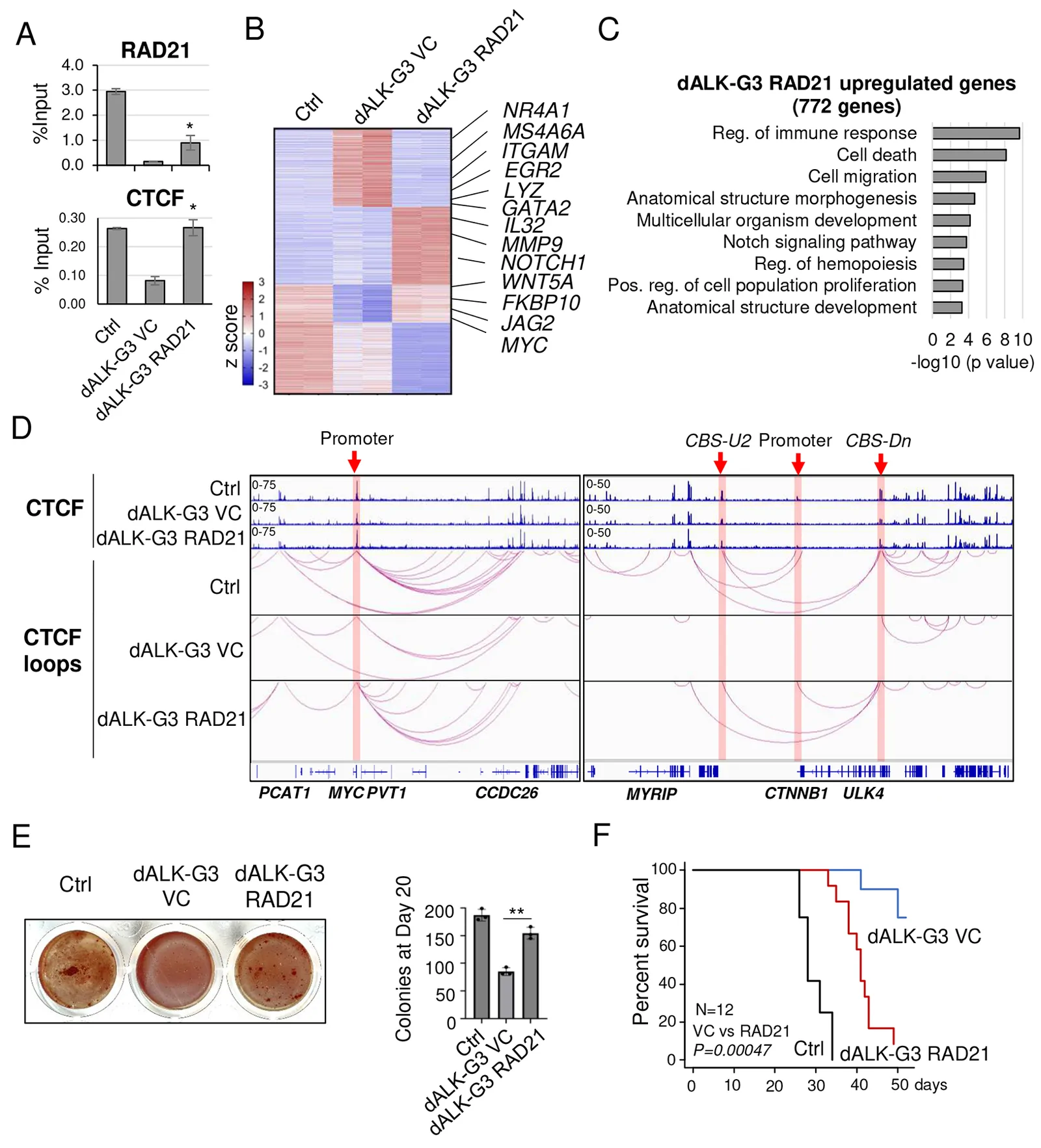
Restoration of RAD21 on the erased G3 m^6^A site on *HOTTIP* lncRNA partially rescues CTCF-driven loops, transcription, and leukemogenesis. **(A)** RIP-qPCR analysis of interactions between *HOTTIP* and RAD21 or CTCF upon restoration of RAD 21 on m^6^A erased G3 site on *HOTTIP.* UV-crosslinked cellular extract was precipitated with anti-RAD21 or anti-CTCF antibody comparing Ctrl, dALK-G3 vector control (dALK-G3 VC) and dALK-G3+dCas13-RAD21 targeting to erased G3 m^6^A site (dALK-G3 RAD21). Purified RNA was detected by qPCR. Data are presented as mean ± SD. *p<0.05 by ANOVA following Bonferroni post hoc test. **(B)** Heatmap of z-score in DEGs upon restoration of RAD21 on m^6^A erased G3 site on *HOTTIP*. RNA-seq was performed on RNAs that were isolated from Ctrl MOLM13 cells or cells expressed dALK-G3 vector control (dALK-G3 VC), or dALK-G3 RAD21 vector. DEGs were selected using FC>1.5, p<0.05. **(C)** Gene ontology analysis of the upregulated 772 genes identified by RNA-seq comparing between the restoration of *HOTTIP*-RAD21 in dALK-G3 cells to dALK-G3 vector control. **(D)** Snapshots of CTCF chromatin binding and corresponding CTCF-mediated chromatin loops at *MYC* and *CTNNB1* loci detected by HiChIP comparing among Ctrl, dALK-G3 VC and dALK-G3 RAD21 MOLM13 cells. Red arrows show CTCF-mediated loop anchors overlapped with promoter and CTCF binding sites of TAD boundaries of *MYC* and *CTNNB1* loci. **(E)** Colony forming assay of targeted expressing RAD21 comparing Ctrl, dALK-G3 VC, dALK-G3/RAD21 MOLM13 cells. Left, Whole well image of colonies at day 20 post-seeding in colony forming assay. Right, quantification of colony number at day 20 post-seeding. **p<0.01 by ANOVA following Bonferroni post hoc test. **(F)** Kaplan-Meier survival curve of NSG mice transplanted with Ctrl, dALK-G3 vector control (VC) or dALK-G3 RAD21 cells. N=12 per group, P=0.00047 RAD21 vs VC by log-rank test followed by Bonferroni post hoc test.

## Highlights:

- High-resolution m^6^A eCLIP-seq defines AML patient-enriched, nucleotide-level m^6^A sites on oncogenic *HOTTIP*.
- Site-specific m^6^A modulates *HOTTIP* dependent enhancer/promoter communication and oncogenic transcription.
- Site-specific erase of m^6^A impaired CTCF-driven TADs and looping leading to mitigation of *MLLr*^+^-driven AML severity.
- Precise recruitment of RAD21 or YTHDF2 to a m^6^A-erased site restores CTCF-driven oncogenic 3D genome.
