# Supplementary material for: Site-specific N6-methyladenosine fine tunes CTCF/cohesin-defined enhancer-promoter interactions and oncogenic transcription program
Source: Res Sq. 2026 Aug 14:rs.3.rs-10010180. Preprint. [Version 1] doi: 10.21203/rs.3.rs-10010180/v1 (PMC13640890; doi:10.21203/rs.3.rs-10010180/v1)
Supplement: Supplementary file 1 [file NIHPPrs10010180v1-supplement-1.pdf]

Figure S1

A

LC-MS/MS ID of *HOTTIP-associated* protein complexes in AML cells (partial list of proteins):

Protein ID	Size (kDa)	Uni No. of Peptides	Function
SMC3	141	13	CTCF/cohesin
CTCF	130	7	
RAD21	71	7	
SMC1A	143	5	
YTHDF2	62	6	
YTHDC1	110	1	m6A reader/ Erase/writer
ALKBH5	44	1	
METTL3	64	7	
METTL14	65	2	
DHX9	141	9	Sensor/regulator of R-loops
PARP1	113	6	
SRSF1	28	4	
RPA1	68	3	
SUPT16H	120	3	
MCM3	91	5	
ASH2L	69	3	MLL/DOT1L
WDR5	37	2	
KMT2A	320	1	
RBBP5	59	1	
DOT1L	185	1	

B

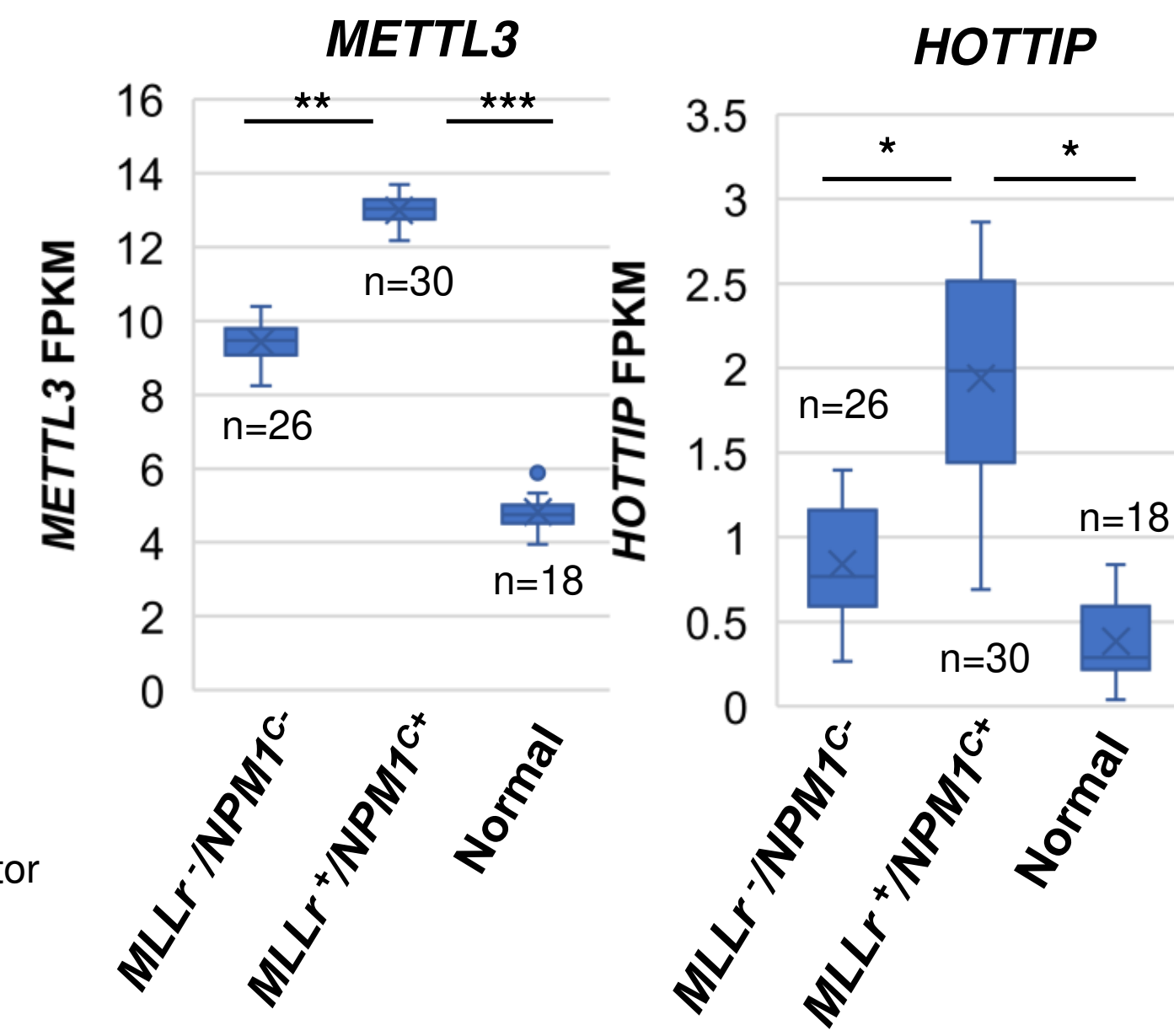

C

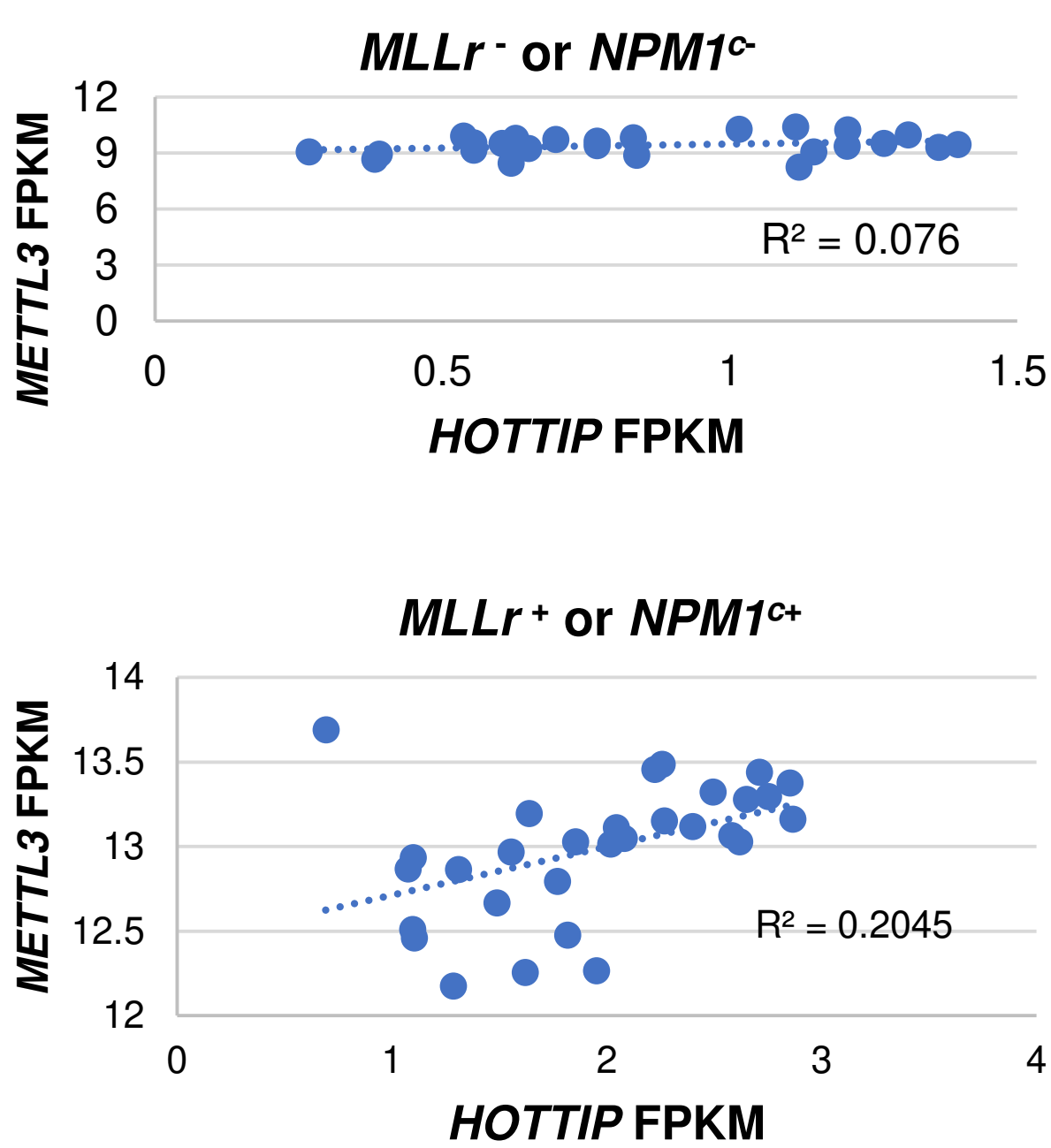

D

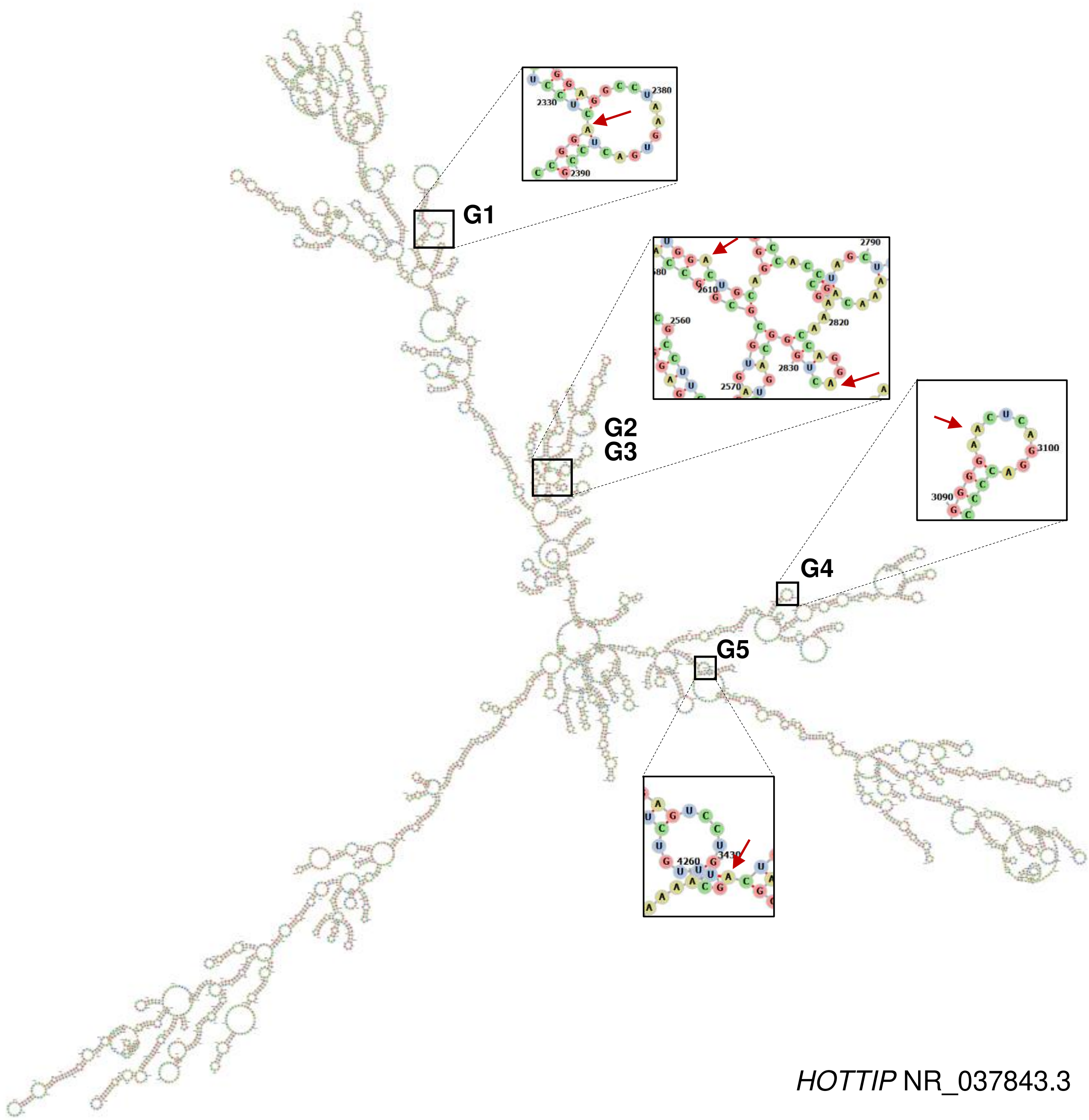

Supplementary Figure 1. Related to Figure 1; *HOTTIP* associates with m^{6}A modifiers/readers and is highly expressed in AML patients.

(A) Partial list of unique peptides identified in *HOTTIP* ChIRP mass spectrometry, but not in *Lacz* ChIRP mass spectorometry (Luo et al., *Mol. Cell.* 2022). Table includes annotated protein IDs, molecular weight (kDa), unique peptide number and functional annotations. **(B)** Analysis from the TCGA-LAML and TARGET-AML patient RNA-seq datasets. FPKM values of *METTL3* and *HOTTIP* of AML patients carrying *MLLr$^+$/NPM1^{C+}* or *MLLr$^-$/NPM1^{C-}*, compared with those of healthy donors. **(C)** Pearson correlation coefficient score is calculated from the FPKM values of *METTL3* and *HOTTIP* in AML patients carrying *MLLr $^+$/NPM1^{C+}* or *MLLr $^-$/NPM1^{C-}* mutations using the RNA-seq data from TCGA-LAML and TARGET-AML datasets. **(D)** Visualization of full length of *HOTTIP* lncRNA secondary structure (NR_037843.3) from RNAfold with default parameters (http://rna.tbi.univie.ac.at/). Minimum free energy is used as fold algorism. Red arrows indicate m^{6}A modification sites at G1-G5.

Figure S2

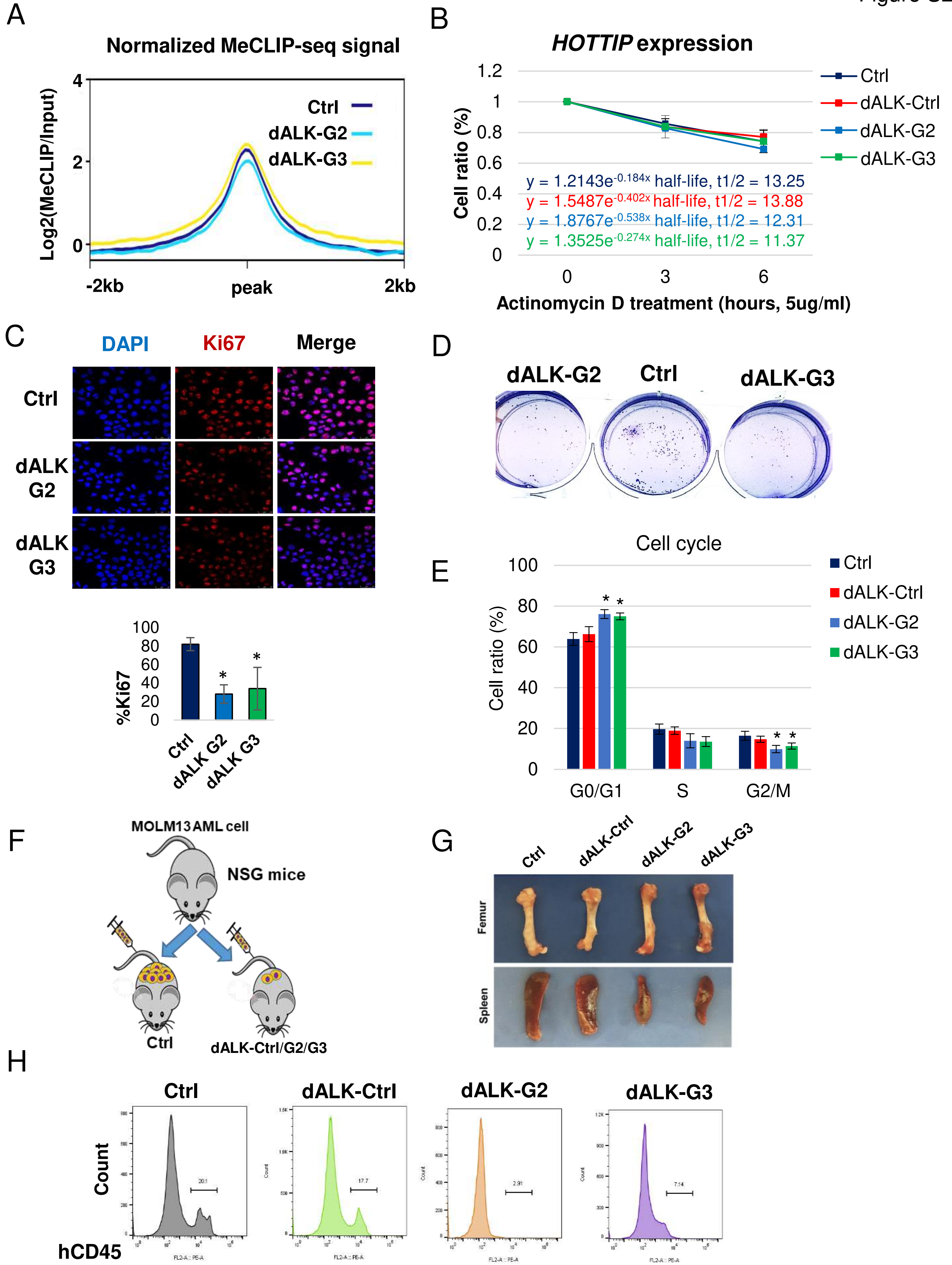

A
Normalized MeCLIP-seq signal
Log2(MeCLIP/Input)
Ctrl
dALK-G2
dALK-G3
-2kb
peak
2kb
B
HOTTIP expression
Cell ratio (%)
Ctrl
dALK-Ctrl
dALK-G2
dALK-G3
y = 1.2143e-0.184x half-life, t1/2 = 13.25
y = 1.5487e-0.402x half-life, t1/2 = 13.88
y = 1.8767e-0.538x half-life, t1/2 = 12.31
y = 1.3525e-0.274x half-life, t1/2 = 11.37
Actinomycin D treatment (hours, 5ug/ml)
C
DAPI
Ki67
Merge
Ctrl
dALK G2
dALK G3
%Ki67
Ctrl
dALK G2
dALK G3
D
dALK-G2
Ctrl
dALK-G3
E
Cell cycle
Cell ratio (%)
Ctrl
dALK-Ctrl
dALK-G2
dALK-G3
G0/G1
S
G2/M
F
MOLM13 AML cell
NSG mice
Ctrl
dALK-Ctrl/G2/G3
G
Ctrl
dALK-Ctrl
dALK-G2
dALK-G3
Femur
Spleen
H
Ctrl
dALK-Ctrl
dALK-G2
dALK-G3
Count
hCD45

I

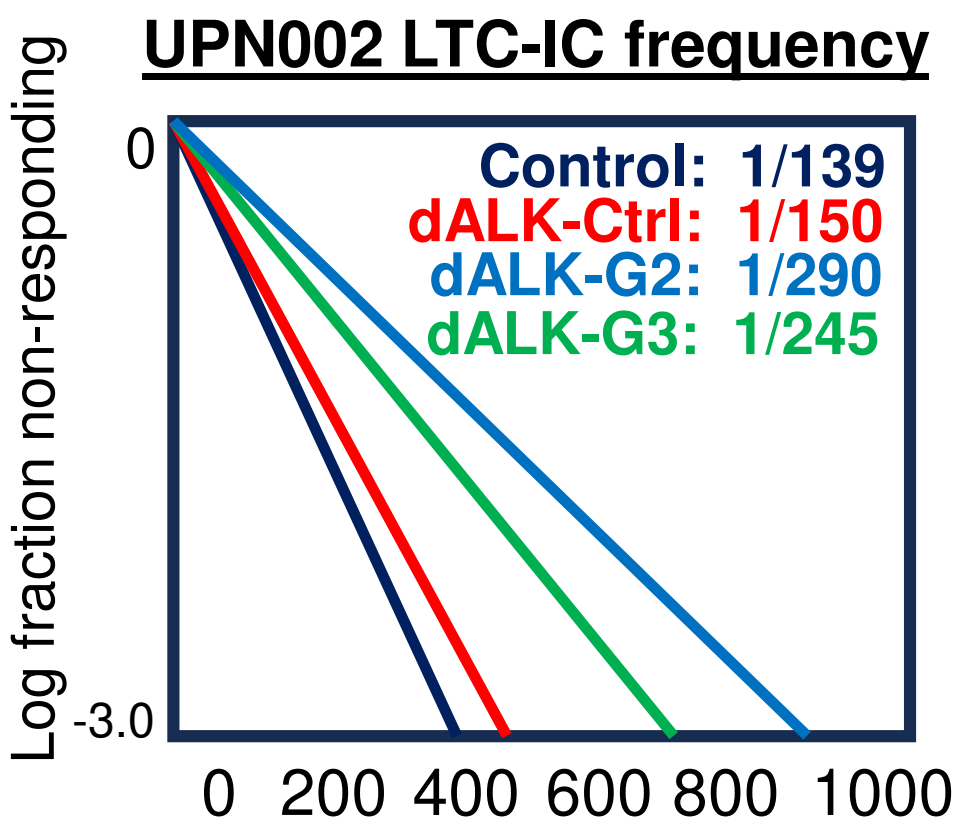

J

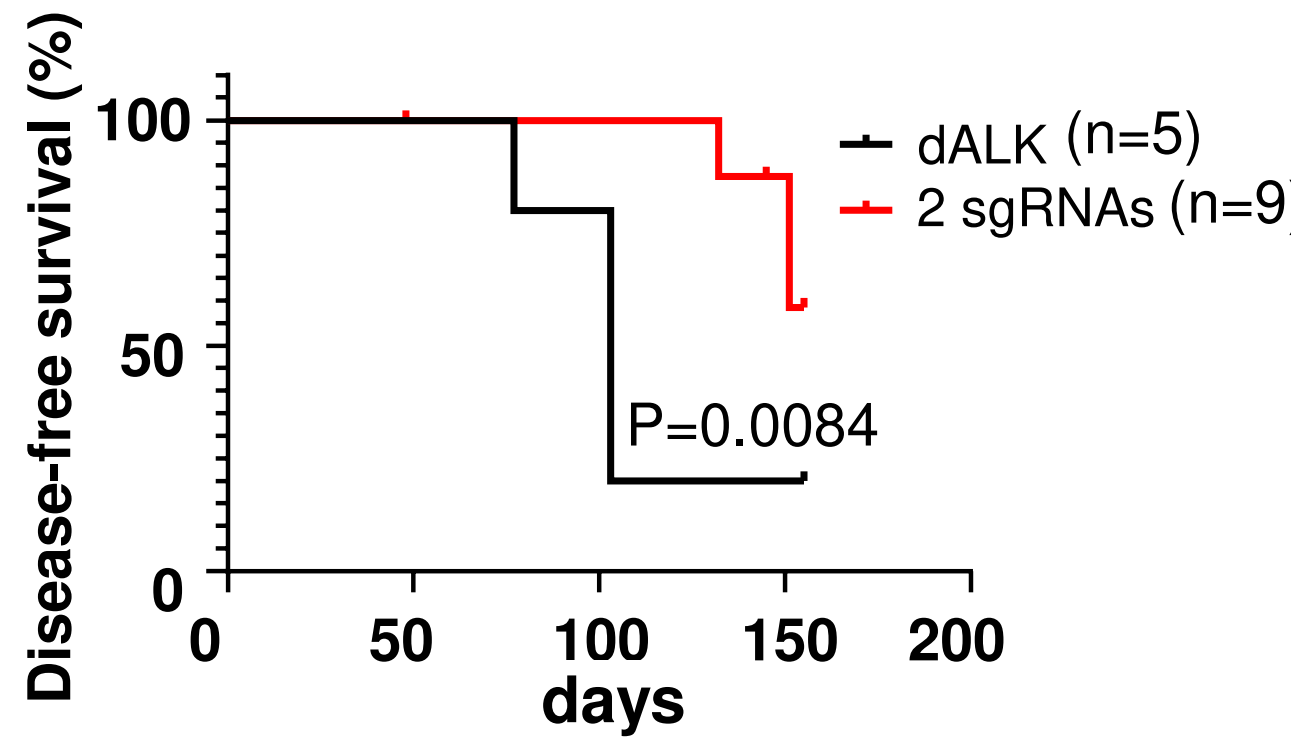

Supplementary Figure 2. Related to Figure 2; Erasing m^{6}A modification of *HOTTIP* RNA impairs LSC activities and prolongs survival of transplanted AML mouse models. (A) Metaplots representing the average normalized m^{6}A MeCLIP signals in the genome-wide regions comparing Ctrl, dALK-G2, or dALK-G3 MOLM13 clones. Signals within ± 2000 bp of the peaks are shown. **(B)** RT-qPCR of *HOTTIP* mRNAs after treatment with actinomycin D. MOLM13 control cells or dCas13-ALKBH5 G2 or G3 cells were treated with actinomycin D (5µg/ml) and harvested at the indicated time. The mRNA abundance at the indicated time point was normalized to non-treated *HOTTIP* mRNA. Error bars denote SD. **(C)** Ki67 immunofluorescence staining image in Ctrl, dALK-G2 and dALK-G3 (Top). Quantification of Ki67 positive MOLM13 cells (Bottom). Data are presented as mean ± SD. *p<0.05, **p<0.01, ***p<0.001 by ANOVA followed Bonferroni post hoc test. **(D)** Frequency of colony formation of dCas13-ALKBH5 G2/G3 cells. MOLM13 control cells or dCas13-ALKBH5 G2 or G3 cells were seeded for 21 days and the colonies were stained with crystal violet. **(E)** Cell cycle analysis of dCas13-ALKBH5 G2 or G3 cells. MOLM13 control or dCas13-ALKBH5 G2 or G3 cells were subjected to flow cytometry, and cell percentage in different cell cycle phases were quantified. Error bars denote standard error of the mean (SEM). $*p < 0.05$ by ANOVA followed Bonferroni post hoc test. **(F)** Schematic representation of transplantation of MOLM13 control or dCas13-ALKBH5 G2 or G3 cells into NSG mice. **(G)** Gross appearance of spleens and femur of representative mice that were transplanted with MOLM13 control cells or dCas13-ALKBH5 G2 or G3 cells. **(H)** hCD45+ cell chimerism in BM of NSG mice receiving MOLM13 control cells or dCas13-ALKBH5 G2 or G3 cells. **(I)** Long term culture-initiating cell (LTC-IC) frequency in AML patient (UPN002) cells expressing control or dCas13-ALKBH5 constructs (dALK-G2, dALK-G3). The frequency of LTC-ICs was calculated using limiting dilution analysis (LDA). **(J)** Kaplan-Meier survival curves of NSG mice transplanted with AML patient cells expressing dCas13-ALKBH5 (dALK) alone, or with both sgRNAs targeting G2 m^{6}A site and G3 m6A site. P-value was calculated by log-rank test followed by Bonferroni post hoc test. n=5 for control group and n=9 for double-sgRNA group.

Figure S3

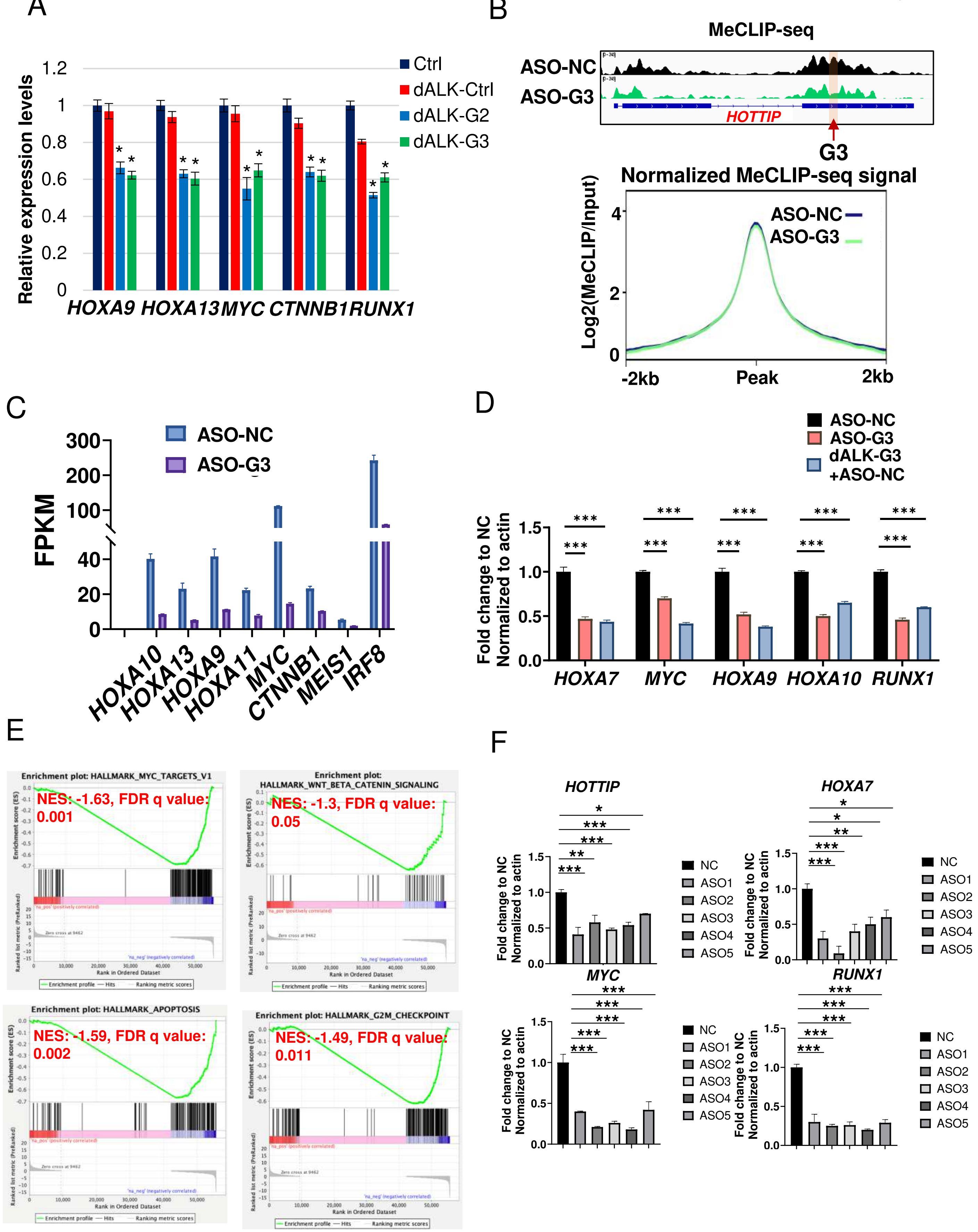

A
Relative expression levels
Ctrl
dALK-Ctrl
dALK-G2
dALK-G3
HOXA9 HOXA13 MYC CTNNB1 RUNX1
B
MeCLIP-seq
ASO-NC
ASO-G3
HOTTIP
G3
Normalized MeCLIP-seq signal
Log2(MeCLIP/Input)
ASO-NC
ASO-G3
-2kb
Peak
2kb
C
FPKM
ASO-NC
ASO-G3
HOXA10 HOXA13 HOXA9 HOXA11 MYC CTNNB1 MEIS1 IRF8
D
ASO-NC
ASO-G3
dALK-G3 +ASO-NC
Fold change to NC Normalized to actin
HOXA7 MYC HOXA9 HOXA10 RUNX1
E
Enrichment plot: HALLMARK_MYC_TARGETS_V1
NES: -1.63, FDR q value: 0.001
Enrichment plot: HALLMARK_WNT_BETA_CATENIN_SIGNALING
NES: -1.3, FDR q value: 0.05
Enrichment plot: HALLMARK_APOPTOSIS
NES: -1.59, FDR q value: 0.002
Enrichment plot: HALLMARK_G2M_CHECKPOINT
NES: -1.49, FDR q value: 0.011
F
HOTTIP
HOXA7
MYC
RUNX1
Fold change to NC Normalized to actin
NC
ASO1
ASO2
ASO3
ASO4
ASO5

Supplementary Figure 3. Related to Figure 3; Erase of m^6A modifications of *HOTTIP* impairs MLL target gene transcription. (A) Expression of *HOTTIP* targets by RT-qPCR analysis. RNAs were isolated from MOLM13 cells transduced with control vector, dCas13-Ctrl, or dCas13-ALKBH5 (G2 or G3) constructs. The mRNA expression levels of *HOTTIP* were normalized to *β-actin* mRNA. Error bars denote SD. $*p < 0.05$ by ANOVA followed Bonferroni post hoc test. **(B)** Top, snapshots of m^6A-eCLIP (MeCLIP)-seq at the *HOTTIP* locus in MOLM13 cells that were treated with either steric blockage ASO targeting G3 m^6A site (ASO-G3) or a non-targeting control (ASO-NC). MeCLIP-seq was carried out using UV crosslinking of the anti-m^6A antibody to the fragmented mRNA. Bottom, metaplots representing the average normalized m^6A MeCLIP signals in the whole genome-wide regions with m^6A signal comparing ASO-NC and ASO-G3. Signals within ± 2000 bp of the peaks are shown. **(C)** FPKM values of target genes from RNA-seq analysis. RNA-seq was performed on RNAs isolated from the MOLM13 cells that were treated with either steric blockage ASO targeting G3 m^6A site (ASO-G3) or a non-targeting control (ASO-NC). **(D)** RT-qPCR validation of the effects of G3-targeting cells on *HOTTIP* targets. MOLM13 control cells or dCas13-ALKBH5 G3 cells were treated with either steric-blockage ASO targeting G3 m^6A site (ASO-G3) or a non-targeting control (ASO-NC). Error bars denote standard error of the mean (SEM). $***p < 0.001$, not significant (two-way ANOVA followed by post-hoc tests). **(E)** Enrichment of genes involved in different pathways and cellular processes upon the treatment of ASO-G3 on *HOTTIP* by GSEA (Gene set enrichment analysis). **(F)** Expression of representative *HOTTIP* targets by RT-qPCR analysis with Gapmer after ASO treatment (*HOTTIP* degradation ASOs). RNAs were isolated from MOLM13 cells and were transfected with 5 Gapmer ASOs individually (ASO1, ASO2, ASO3, ASO4, ASO5) targeting for *HOTTIP* degradation as positive controls. The mRNA expression levels were normalized to *β-actin* mRNA. Error bars denote standard SEM. $*p < 0.05$, $**p < 0.01$, $***p < 0.001$. n.s., not significant (One-way ANOVA followed by post-hoc tests).

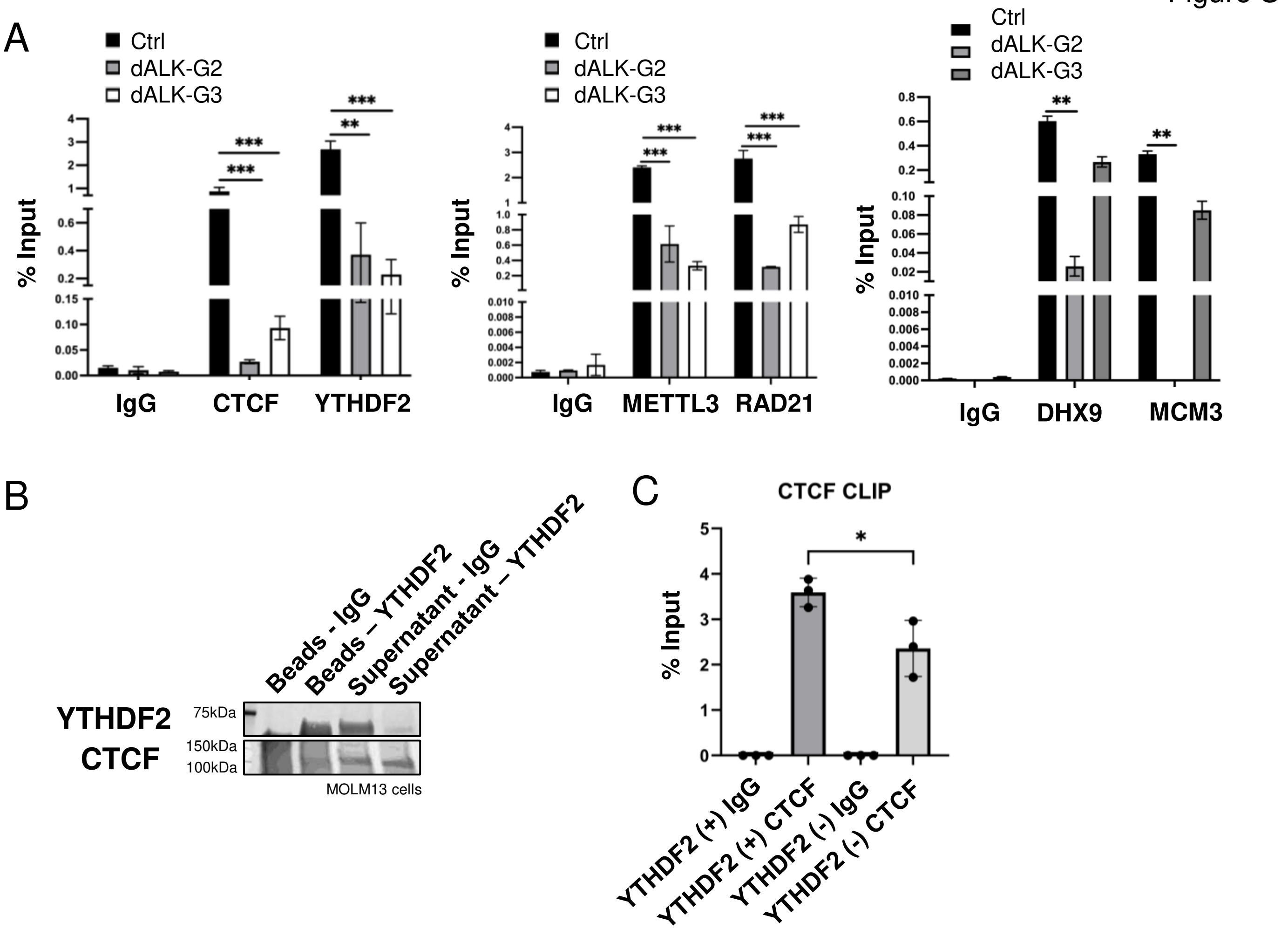

Supplementary Figure 4. Related to Figure 4; Erase of m^{6}A modifications of *HOTTIP* disrupted *HOTTIP* interactions with CTCF/cohesin complex, m^{6}A writer/readers and R-loop sensors/regulators. (A) CLIP-qPCR experiments to examine binding of indicated m^{6}A writers, readers or chromatin organizers to *HOTTIP* RNA. CLIP experiments were performed using the indicated antibodies on MOLM13 control cells or dCas13-ALKBH5 G2 or G3 cells. IgG was used as a negative control for CLIP experiments. Error bars denote SD. ∗p < 0.05, ∗∗p < 0.01, ∗∗∗p < 0.001, n.s., not significant by ANOVA followed Bonferroni post hoc test. **(B)** Western blot to confirm YTHDF2 proteins are depleted in the supernatant after incubation with IgG or anti-YTHDF2 antibody in MOLM13 cells. CTCF protein level in the supernatant is shown as a control as it was not affected by anti-YTHDF2 incubation. Supernatant is used for the following CTCF CLIP assay in Figure S4C. **(C)** CTCF CLIP-qPCR shows that the CTCF-*HOTTIP* interaction was impaired after YTHDF2 depletion. CTCF CLIP-qPCR after YTHDF2 depletion was performed using the MOLM13 supernatant depleted YTHDF2. Data are presented as mean ± SD. *p<0.05 by Student t-test.

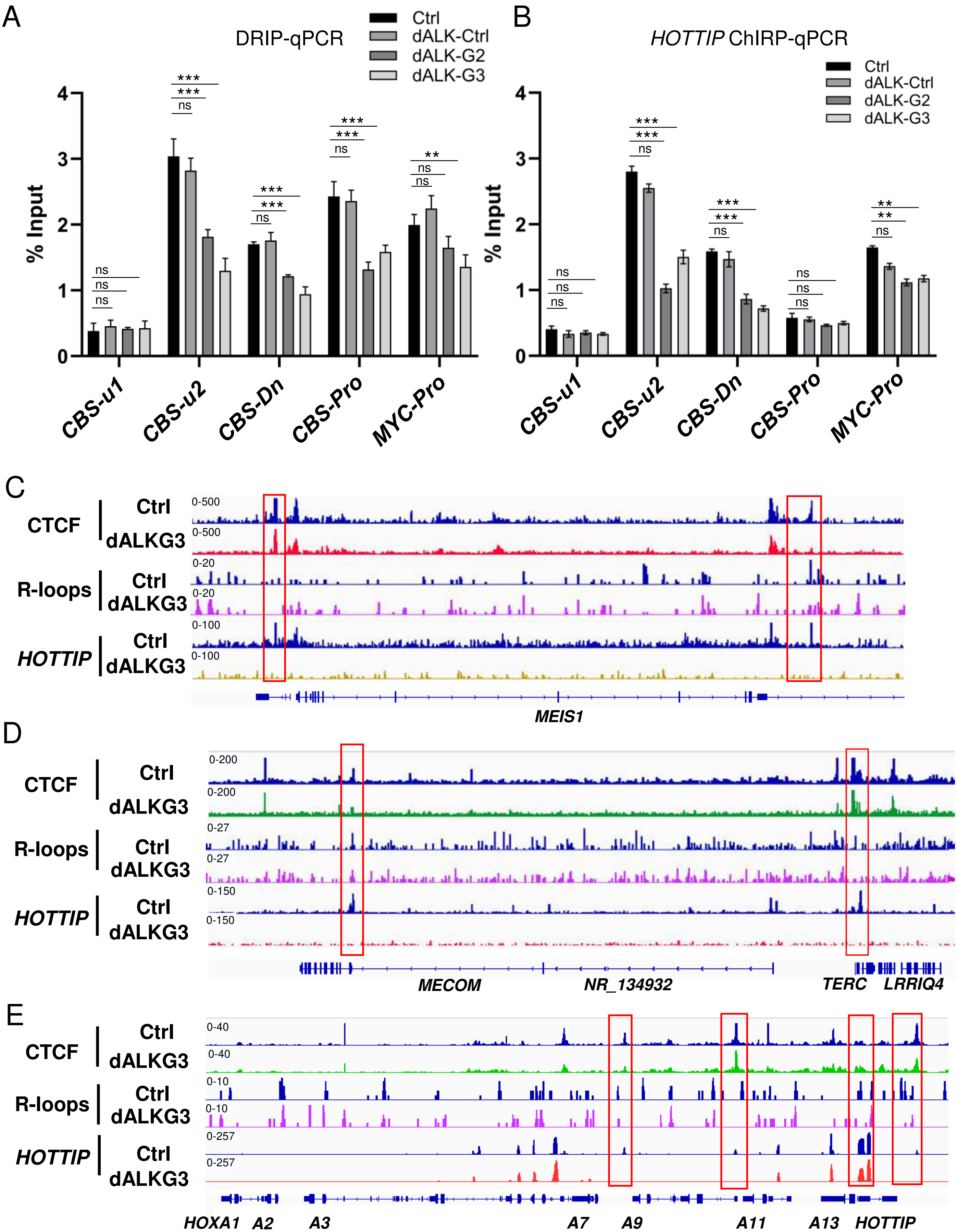
A
DRIP-qPCR
Ctrl
dALK-Ctrl
dALK-G2
dALK-G3
% Input
CBS-u1
CBS-u2
CBS-Dn
CBS-Pro
MYC-Pro
B
HOTTIP ChIRP-qPCR
C
CTCF
R-loops
HOTTIP
Ctrl
dALKG3
MEIS1
D
MECOM
NR_134932
TERC
LRRIQ4
E
HOXA1
A2
A3
A7
A9
A11
A13
HOTTIP

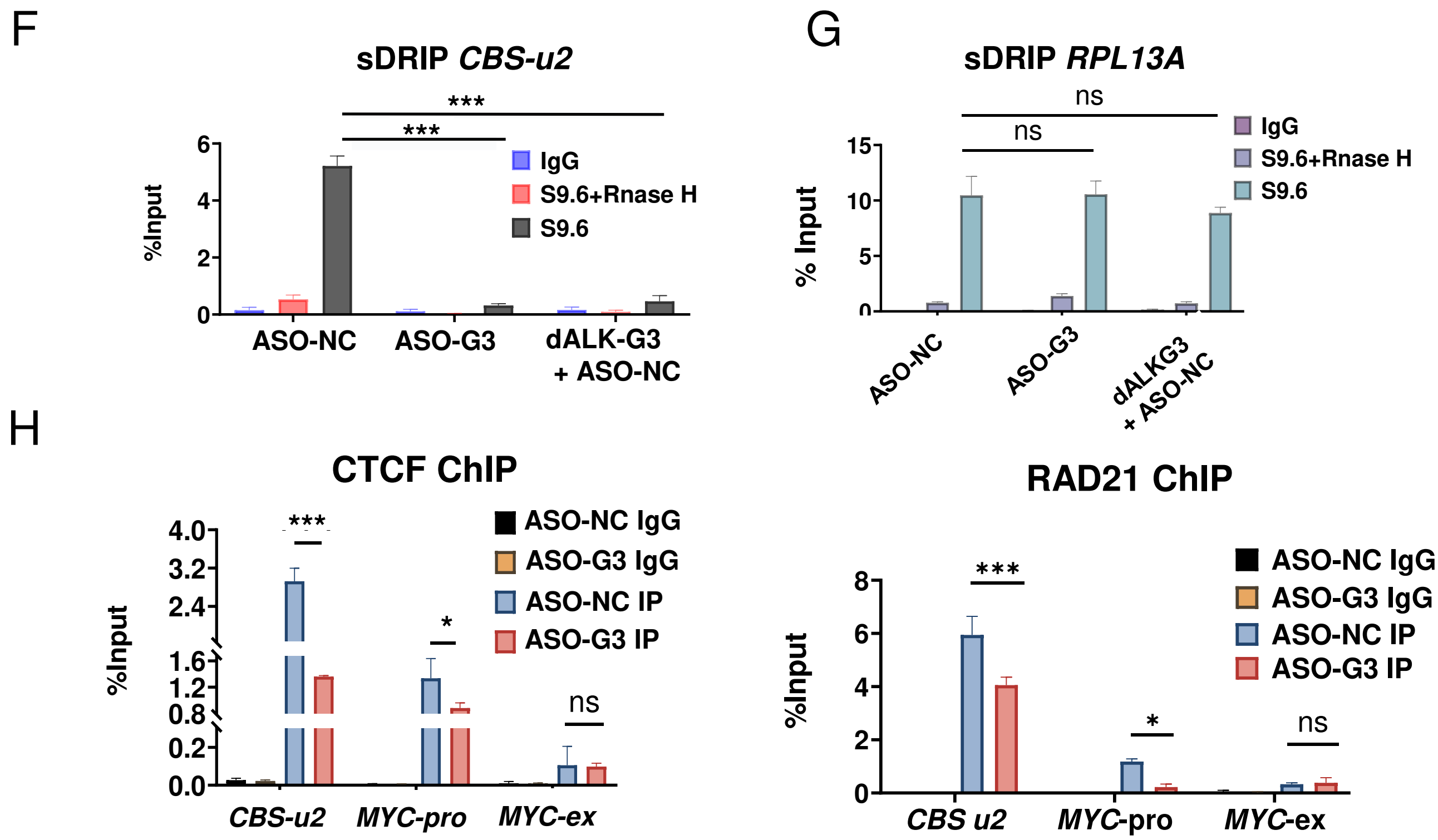

Supplementary Figure 5. Related to Figure 5; m^{6}A in *HOTTIP* regulates R-loop formation at *HOTTIP*/CTCF co-bounded sites. (A) DRIP-qPCR to examine R-loops at *HOTTIP* and CTCF co-bound sites (*CBS-u1, CBS-u2, CBS-Dn, CBS-Pro, MYC-Pro*) in Ctrl, dALK-Ctrl, dALK-G2 and dALK-G3. Data are presented as mean ± SD. *p<0.05, **p<0.01, ***p<0.001, ns, not significant, by ANOVA followed Bonferroni post hoc test. **(B)** *HOTTIP* ChIRP-qPCR at *HOTTIP* and CTCF co-binding sites (*CBS-u1, CBS-u2, CBS-Dn, CBS-Pro, MYC-Pro*) comparing *HOTTIP* chromatin binding in Ctrl, dALK-Ctrl, dALK-G2 and dALK-G3. Data are presented as mean ± SD. *p<0.05, **p<0.01, ***p<0.001, ns, not significant by ANOVA followed Bonferroni post hoc test. **(C, D and E)** Snapshots of CTCF binding, R-loops peaks and *HOTTIP* binding at *MEIS1* **(C)**, *MECOM* **(D)** and *HOXA* **(E)** loci compared Ctrl and dALK-G3 MOLM13 cells. Red box highlighted regions indicate CTCF/*HOTTIP* co-binding sites at *MEIS1*, *MECOM* or *HOXA* loci. **(F and G)** sDRIP-qPCR to examine R-loop formation at *CBS-u2* **(F)** and *RPL13A* **(G)** loci. sDRIP experiments were performed using S9.6 antibody in MOLM13 cells treated either with ASO targeting G3 site or a non-targeting negative control (ASO-NC). IgG and RNAse H treated samples were used as negative controls. Error bars denote SEM. ***p < 0.001, n.s., not significant (two-way ANOVA followed by post-hoc tests). **(H)** CTCF or RAD21-ChIP-qPCR to examine binding of CTCF (Left panel) or RAD21 (Right panel) to the indicated *HOTTIP* targets loci Upon blockage of G3 m^{6}A. *MYC* exon locus served as a non-target control. ChIP experiments were performed using anti-CTCF or anti-RAD21 antibody in MOLM13 cells treated either with ASO targeting G3 site or a non-targeting negative control (ASO-NC). IgG was used as a negative control for ChIP experiments. Error bars denote SEM. *p < 0.05, **p < 0.01, ***p < 0.001, n.s., not significant (two-way ANOVA with post hoc test).

Figure S6

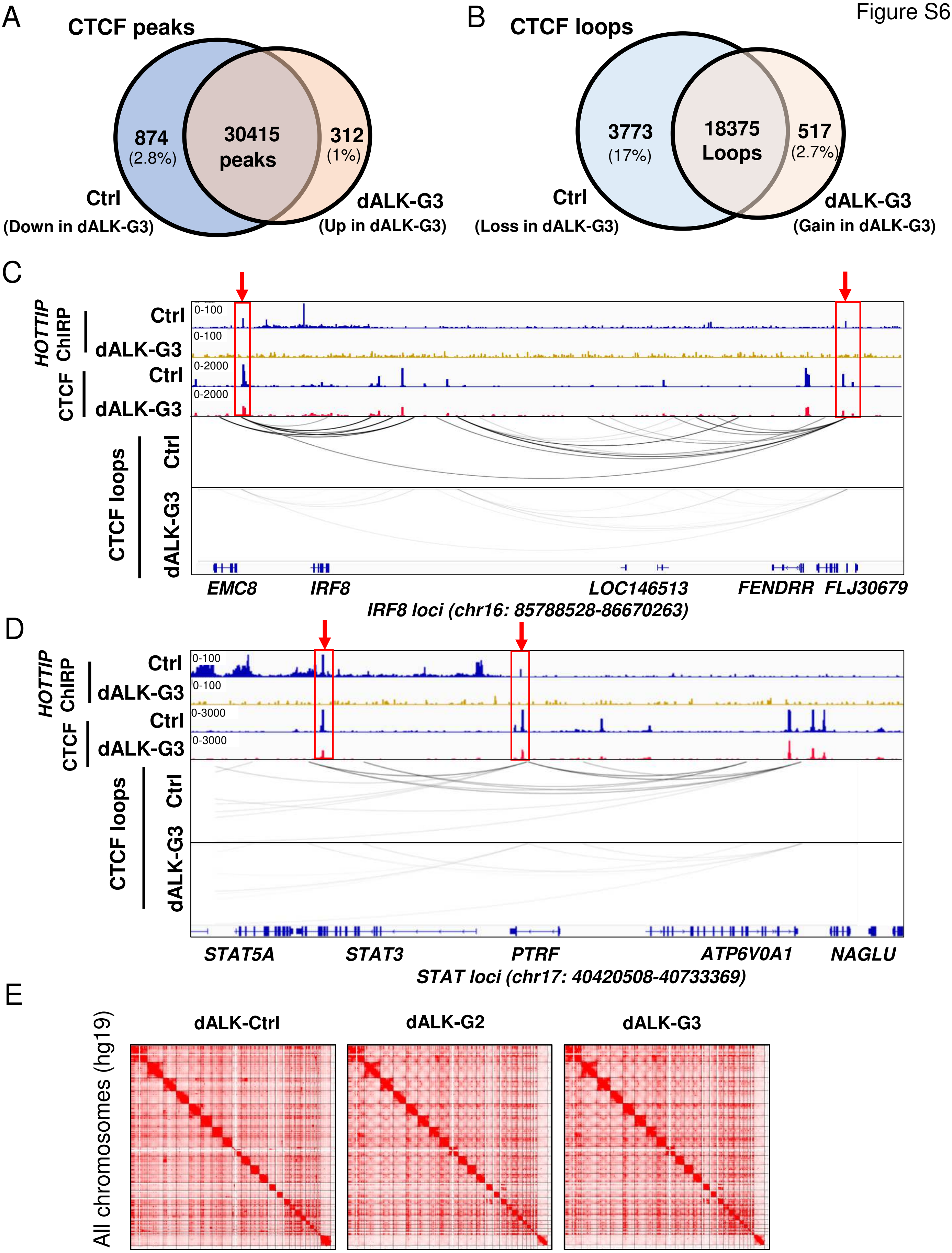
A
CTCF peaks
874
(2.8%)
30415
peaks
312
(1%)
Ctrl
(Down in dALK-G3)
dALK-G3
(Up in dALK-G3)
B
CTCF loops
3773
(17%)
18375
Loops
517
(2.7%)
Ctrl
(Loss in dALK-G3)
dALK-G3
(Gain in dALK-G3)
C
HOTTIP ChIRP
Ctrl
dALK-G3
CTCF
Ctrl
dALK-G3
0-100
0-100
0-2000
0-2000
CTCF loops
Ctrl
dALK-G3
EMC8
IRF8
LOC146513
FENDRR
FLJ30679
IRF8 loci (chr16: 85788528-86670263)
D
HOTTIP ChIRP
Ctrl
dALK-G3
CTCF
Ctrl
dALK-G3
0-100
0-100
0-3000
0-3000
CTCF loops
Ctrl
dALK-G3
STAT5A
STAT3
PTRF
ATP6V0A1
NAGLU
STAT loci (chr17: 40420508-40733369)
E
All chromosomes (hg19)
dALK-Ctrl
dALK-G2
dALK-G3

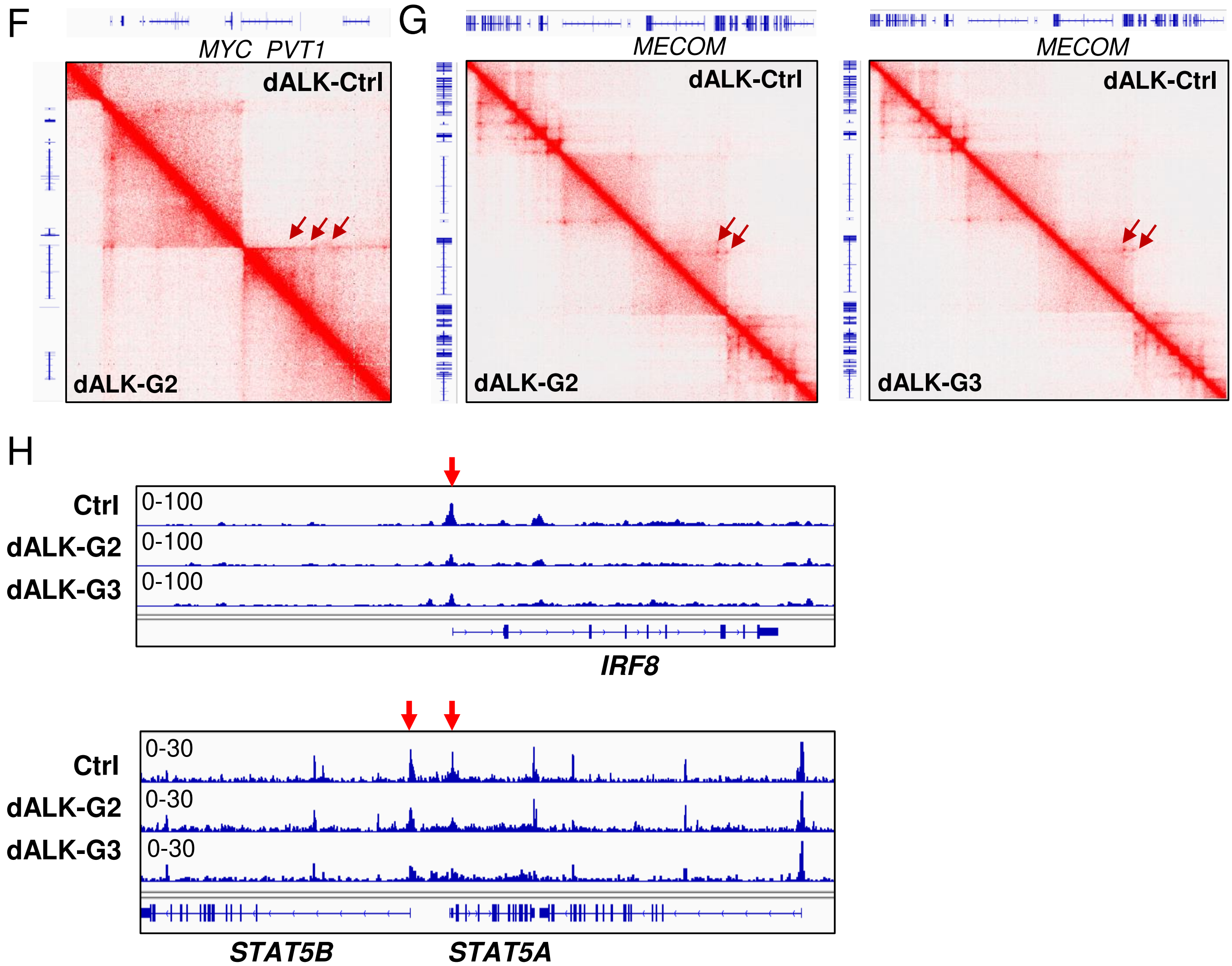

F
MYC PVT1
dALK-Ctrl
dALK-G2
G
MECOM
dALK-Ctrl
dALK-G2
MECOM
dALK-Ctrl
dALK-G3
H
Ctrl
dALK-G2
dALK-G3
0-100
0-100
0-100
IRF8
Ctrl
dALK-G2
dALK-G3
0-30
0-30
0-30
STAT5B
STAT5A

Supplementary Figure 6. Related to Figure 6; Specific targeting G3 m^6A site loses a subset of CTCF-mediated chromatin loops and chromatin accessibility at *HOTTIP* target loci.

(A) Venn diagram of altered CTCF binding from CTCF HiChIP-seq comparing Ctrl and dALK-G3 cells. CTCF peaks are called using macs2 callpeak with $q<0.001$. Differential peaks are identified using bedtools intersect. **(B)** Venn diagram of gain or lost CTCF-mediated chromatin loops from CTCF-HiChIP upon erase of G3 m^6A modification in dALK-G3 cells. Differential loops are called by HiCCUPSdiff in JuicerTools with 5k, 10k, 25k resolution. **(C and D)** Snapshots of *HOTTIP* binding, CTCF binding and CTCF-mediated chromatin loops at *IRF8* **(C)** and *STAT5* **(D)** loci. Red box highlighted regions indicate *HOTTIP*/CTCF co-binding sites at TAD boundaries of *IRF8* or *STAT5* locus. **(E)** HiC-seq interaction map at all chromosomes (hg19) in dALK-Ctrl, dALK-G2 and dALK-G3 showing that global chromatin interaction is not changed by targeting specific m^6A sites. **(F and G)** HiC-seq interaction maps at *MYC* **(F)** and *MECOM* **(G)** loci comparing dALK-Ctrl, dALK-G2, or dALK-G3 cells. HiC interaction matrix is normalized by the Knight-Ruiz (KR) matrix balancing. Red arrows indicate reduced HiC signals upon erase of m^6A at G3 site on *HOTTIP*. **(H)** Snapshots of ATAC-seq tracks at *IRF8* (top) and *STAT5A/B* (bottom) loci. Red arrows indicate reduced ATAC-seq signal at promoter regions comparing Ctrl, dALK-G2 or dALK-G3.

Figure S7

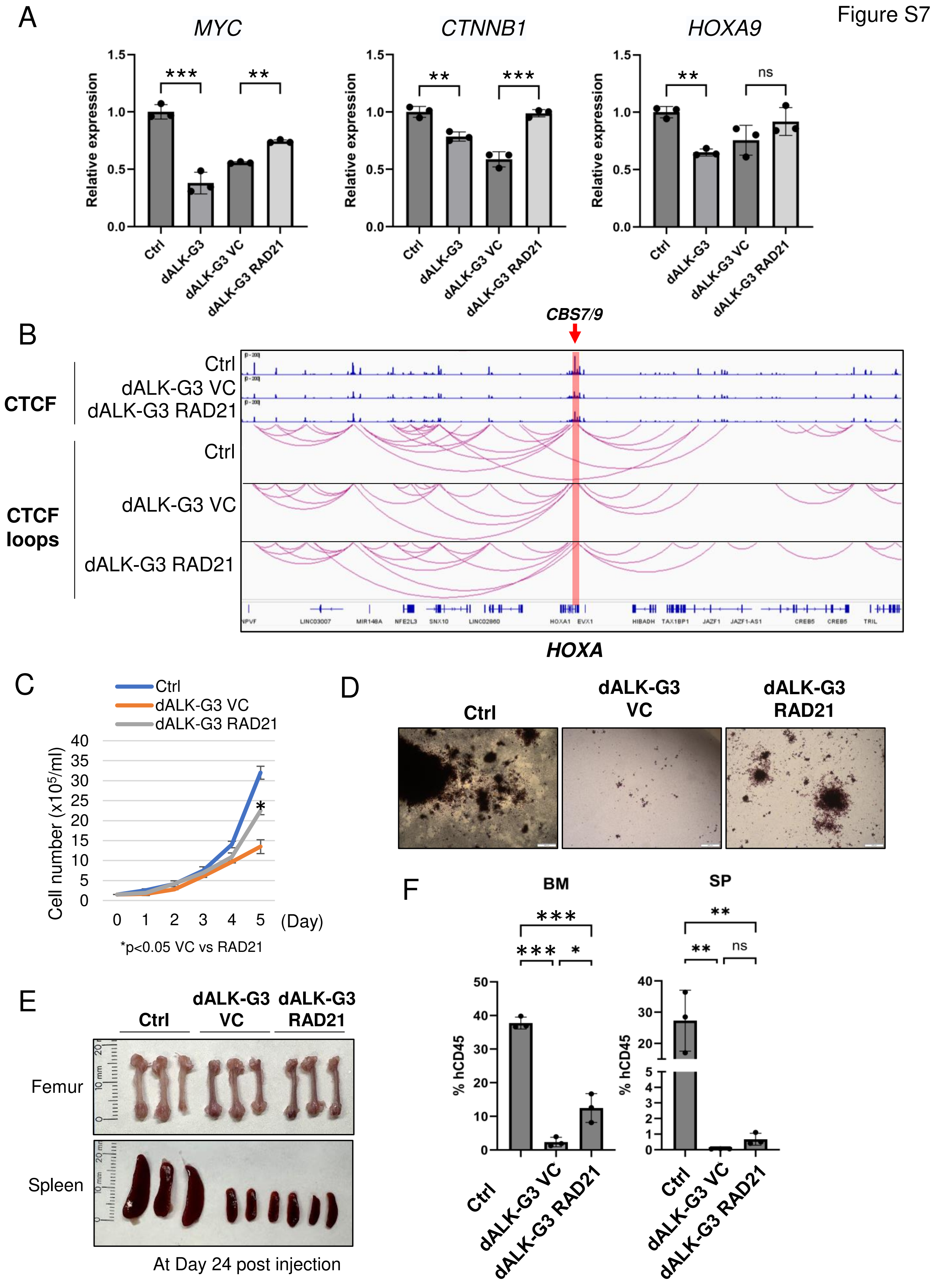

A
MYC
CTNNB1
HOXA9
Relative expression
1.5
1.0
0.5
0.0
***
**
ns
Ctrl
dALK-G3
dALK-G3 VC
dALK-G3 RAD21
B
CBS7/9
CTCF
Ctrl
dALK-G3 VC
dALK-G3 RAD21
CTCF loops
NPVF
LINC03007
MIR148A
NFE2L3
SNX10
LINC02860
HOXA1
EVX1
HIBADH
TAX1BP1
JAZF1
JAZF1-AS1
CREB5
TRIL
HOXA
C
Ctrl
dALK-G3 VC
dALK-G3 RAD21
Cell number (x10^5/ml)
40
35
30
25
20
15
10
5
0
*
0 1 2 3 4 5 (Day)
*p<0.05 VC vs RAD21
D
Ctrl
dALK-G3 VC
dALK-G3 RAD21
E
Ctrl
dALK-G3 VC
dALK-G3 RAD21
Femur
Spleen
At Day 24 post injection
F
BM
SP
***
***
*
**
**
ns
% hCD45
50
40
30
20
10
0
5
4
3
2
1
Ctrl
dALK-G3 VC
dALK-G3 RAD21

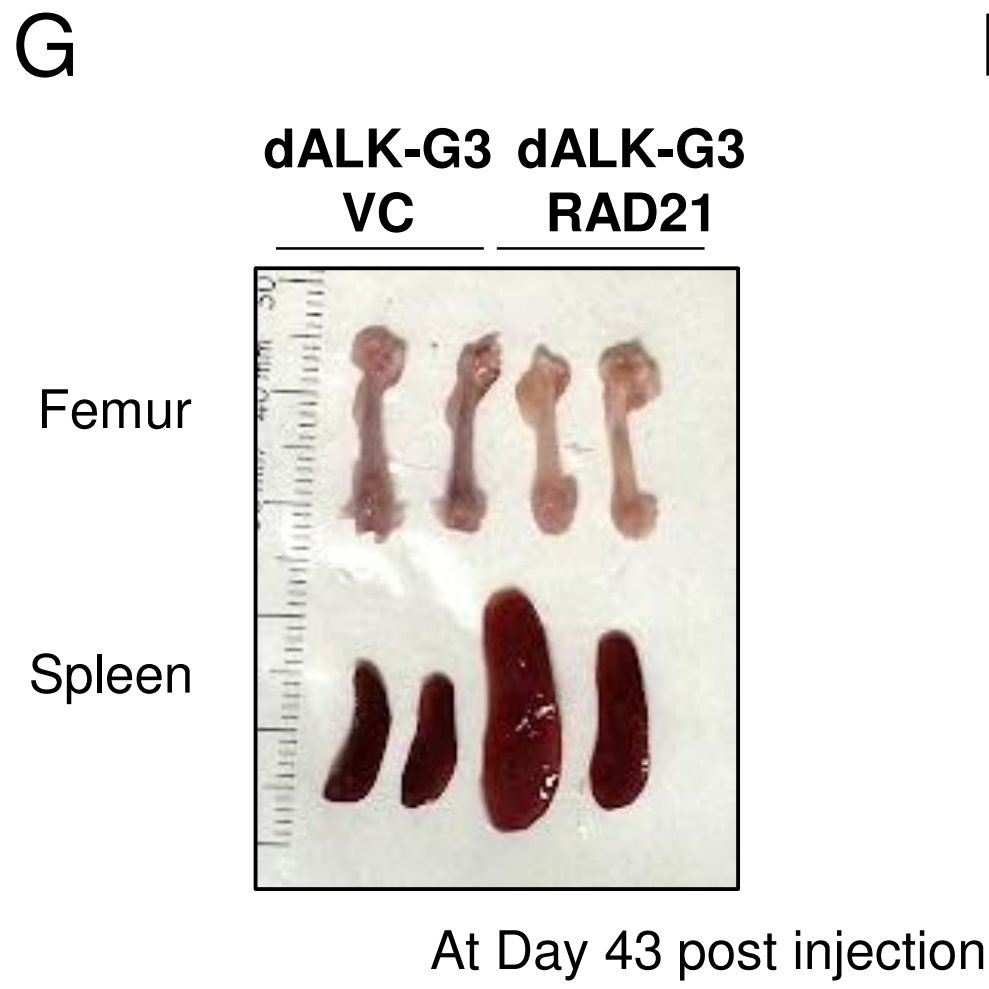

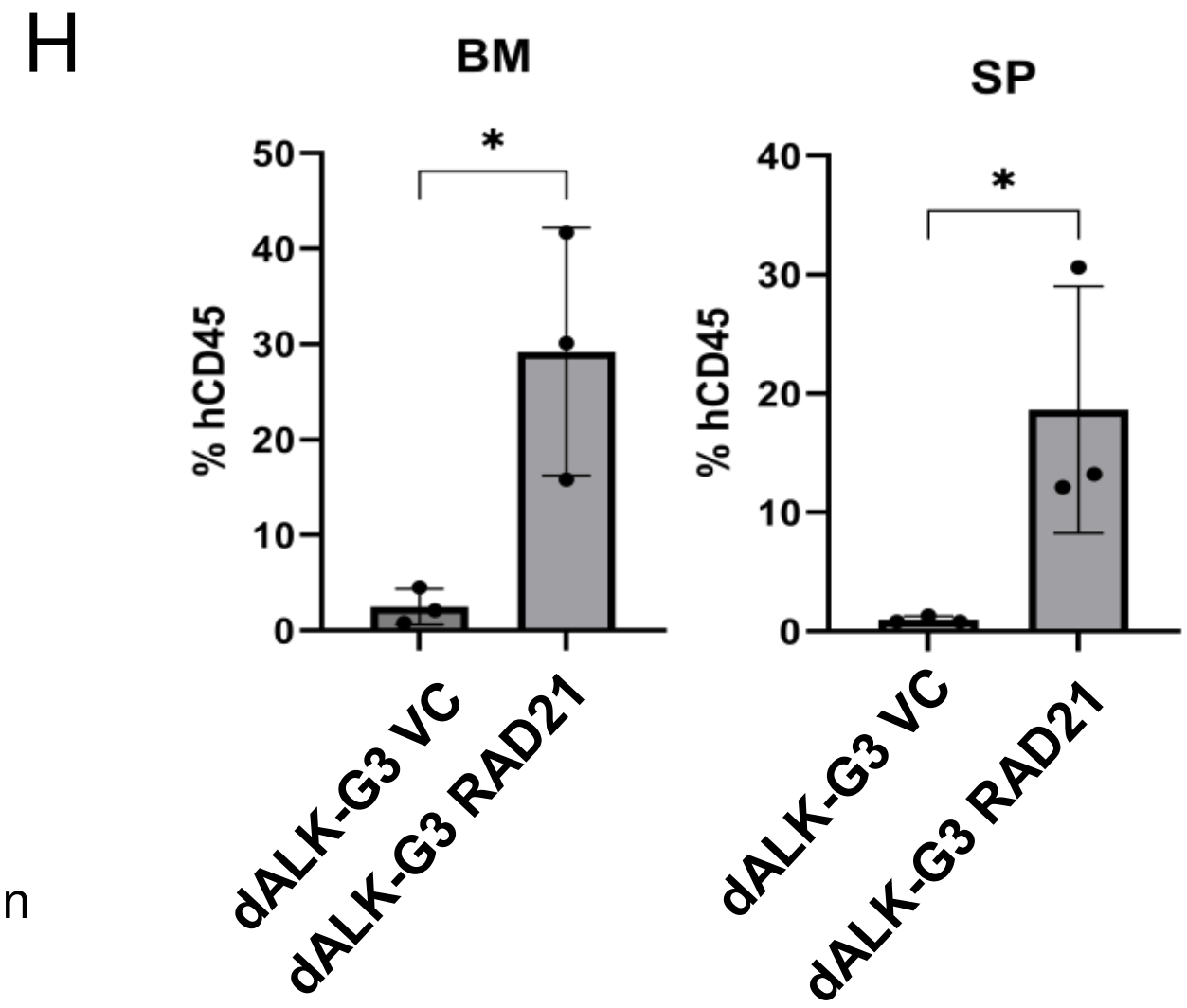

Supplementary Figure 7. Related to Figure 7; Restoration of *HOTTIP*-RAD21 in dALK-G3 cells partially rescues leukemic progression and MYC expression by re-forming CTCF-mediated chromatin loops.

(A) RT-qPCR examined expression levels of *MLLr* targets, *MYC, CTNNB1, and HOXA9* in Ctrl, dALK-G3, dALK-G3 VC, dALK-G3 RAD21 cells. Data are represented as mean ± SD. *p<0.05, **p<0.01, ***p<0.001 by ANOVA followed Bonferroni post hoc test. **(B)** Snapshot of CTCF binding and CTCF-mediated loops from CTCF HiChIP at *HOXA* locus comparing Ctrl, dALK-G3 VC, dALK-G3 RAD21. Red highlight indicates CTCF binding site between *HOXA7* and *HOXA9* (*CBS7/9*). **(C)** Cell proliferation curve of Ctrl, dALK-G3 VC and dALK-G3 RAD21 MOLM13 cells for 5 days. Data are presented as mean ± SD. *p<0.05 by ANOVA followed Bonferroni post hoc test. **(D)** Representative colony images at day 20 post-seeding in colony forming assay. White bar indicates 50μm. **(E)** Gross image of spleen and femur from Ctrl-, dALK-G3 VC-, or dALK-G3 RAD21 MOLM13 cells-transplanted NSG mice at day 24 post injection at the endpoint of Ctrl-transplanted NSG mice. **(F)** Quantitation of hCD45 chimerism in bone marrow cells and spleen cells at day 24 post injection. Data are presented as mean ± SD. *p<0.05, **p<0.01, ***p<0.001 by ANOVA followed Bonferroni post hoc test. **(G)** Gross image of spleen and femur from dALK-G3 VC- or dALK-G3 RAD21 MOLM13 cells-transplanted NSG mice at day 43 post injection, when is the endpoint of dALK-G3 RAD21-transplanted NSG mice. **(H)** Quantitation of hCD45 chimerism in bone marrow cells and spleen cells at day 43 post injection. Data are presented as mean ± SD. *p<0.05 by Student t-test.
